# Supplementary material for: Cost-effectiveness of a combined classroom curriculum and parental intervention: economic evaluation of data from the Steps Towards Alcohol Misuse Prevention Programme cluster randomised controlled trial
Source: BMJ Open. 2019 Jul 2;9(7):e027951. doi: 10.1136/bmjopen-2018-027951 (PMC6609141; doi:10.1136/bmjopen-2018-027951)
Supplement: Supplementary file 1 [file bmjopen-2018-027951supp001.pdf]

Online Supplementary File Cost-effectiveness of a combined classroom curriculum and parental intervention: economic evaluation of data from the Steps Towards Alcohol Misuse Prevention Programme (STAMPP) cluster randomised controlled trial (Agus et al).

Costing the intervention

Stage 1 resources in STAMPP covered; recurring costs associated with printing/copying the intervention materials (teacher manual, pupil work book, parent/guardian information sheets, CD-ROMs) and the delivery of teacher training (trainer fees, venue, catering and teacher cover). No costs associated with the purchasing and licensing of the STAMPP intervention are foreseen and so are not included here. Stage 2 resources covered recurring costs associated with teacher time input for delivery of Phase 1 and Phase 2 of the programme. The cost of teaching time was included, even though the intervention was delivered within normal working hours and so would not have been an extra cost to the public sector. In doing so we captured the opportunity cost of the benefits to pupils which were foregone by STAMPP being delivered instead of other lessons.

Resources used during stages 1 and 2 were recorded retrospectively by the project team and costs were obtained from invoices when available. The costs associated with printing the intervention materials were not available as these resources were present at the start of the trial. We therefore obtained printing quotations and deflated it to the appropriate price year using the Consumer Price Index.[1] The Local Authority in Glasgow/Inverclyde covered all the costs associated with the training of the teachers including teacher cover, venue and catering and so these were also not available. We therefore used the costs that were available for NI and applied it to Scotland. The majority of teachers delivering the intervention were at a junior level, thus we used point 3 on the Main Pay Scale[2] including 24% for employers' costs.

Stage 1 costs were added together to get a total cost. Stage 2 costs relating to the delivery of the intervention at a class level were multiplied by the total number of classes that received intervention to get a total cost. This was estimated by dividing the number of pupils receiving the intervention at baseline (n=5749) by 30, which was used as an estimate of the maximum number of pupils per class. Stage 2 costs relating to the delivery of the parental component were calculated by multiplying the number of schools by the costs to deliver one session. Stage

1 and Stage 2 costs were totalled and divided by the number of pupils receiving the intervention at baseline to obtain the mean cost per pupil.

1. Office for National Statistics. (2015). CPI annual percentage change: 1989 to 2015. Retrieved from http://www.ons.gov.uk/ons/datasets-and-tables/data-selector.html?cdid=D7G7&dataset=mm23&table-id=1.2 (accessed Dec 9 2015)
2. Department of Education Northern Ireland. Circular 2014/13 - Teachers' pay and allowances from 1 September 2013. 2014. Available from https://www.deni.gov.uk/publications/circular-201413-teachers-pay-and-allowances-1-september-2013 (accessed Dec 9 2015)

Table S1 Breakdown of missing cost and/outcome data by group

Missing data	Intervention n=2190 [%]	Education as normal n=2322 [%]	Total n=4512 [%]
Outcome	1219 [57]	1286 [55]	2505 [56]
Cost	2123 [97]	2256 [97]	4379 [97]
Both	1152 [53]	1220 [53]	2372 [53]

Table S2 Intervention resource use and costs

**Resource use**	**Unit cost (£)**	**Number of units**	**Total cost (£)**	**Cost details**
**Stage 1 Planning and preparation for delivery**				
**Intervention materials**				
Phase 1 Teacher Manual	n/a	145	240	16 pages
Phase 2 Teacher Manual	n/a	145	293	22 pages
Phase 1 Pupil Workbook	n/a	5749	2623	20 pages
Phase 2 Pupil Workbook	n/a	5749	2623	20 pages
Parent & Guardian Information Sheet	n/a	5749	255	single-sided page
Parent & Guardian Information Sheet (follow up)	n/a	5749	255	single-sided page
Phase 1 CD-ROM	1.40	145	203	cost of duplication
Phase 2 CD-ROM	1.40	145	203	cost of duplication
**Training of the teachers (Phase 1)**				

Northern Ireland				
Training	500.00	3	1500	Training delivered by a non-government organisation facilitator (£500 per day)
Teachers	161.79	107	17312	Based on 1 day (6.5 hours) teaching cover for a junior teacher at £24.89/hour
Location & associated cost (e.g. room hire)	25.00	107	2675	Based on local hotel costs including food & refreshments per head
Scotland				
Training	500.00	2	1000	Training delivered by a non-government organisation facilitator (£500 per day)
Teachers	161.79	38	6148	Based on 1 day (6.5 hours) teaching cover for a junior teacher at £24.89/hour
Location & associated cost (e.g. room hire)	25.00	38	950	Local hotel costs including food & refreshments (£25/head)
**Training of the teachers (Phase 2)**				
Northern Ireland				
Training sessions	500.00	3	1500	Training delivered by a non-government organisation facilitator (£500 per day)
Teachers	24.89	83	2066	based on 1 day (6.5 hours) teaching cover for a junior teacher
Location & associated cost (e.g. room hire)	28.78	83	2389	Local hotel costs including food & refreshments (£28.78/head)
Scotland				
Trainer	500.00	1	500	Training delivered by a non-government organisation facilitator (£500 per day)
Teachers	24.89	38	946	based on 1 day (6.5 hours) teaching cover for a junior teacher
Location & associated cost (e.g. room hire)	28.78	38	1094	Local hotel costs including food & refreshments (£28.78/head)
**Stage 2 Delivery**				
Phase 1 Teacher time input	14.52	6	87	Based on 35 minute lessons
Phase 2 Teacher time input	14.52	4	58	Based on 35 minute lessons

Parental component delivery	250.00	53	13250	Delivered by a non-government organisation facilitator (£500 per day)

Use of public sector services by all pupils with available data at each of the time points.

Table S3 Public sector service by treatment arm from 6 to 12 months. Values are number (percentages) of pupils using the service and mean (SD) use.

Service	Intervention (n=4189)		Education as normal (n=4037)	
	**Number using the service (%)**	**Mean (SD)**	**Number using the service (%)**	**Mean (SD)**
**Education**				
School nurse	1792 (42.8)	0.9 (3.0)	1518 (37.6)	0.9 (3.4)
School counsellor/guidance teacher	642 (15.3)	0.5 (3.5)	646 (16.0)	0.4 (1.8)
Educational psychologist	71 (1.7)	0.0 (0.8)	84 (2.1)	0.0 (0.4)
Education welfare officer/ home-school liaison officer	59 (1.4)	0.0 (0.4)	89 (2.2)	0.0 (0.9)
**Health**				
GP surgery visit	2033 (48.5)	1.1 (5.0)	1791 (44.4)	0.9 (1.8)
GP out of hours	521 (12.4)	0.2 (0.9)	423 (10.5)	0.2 (0.8)
Nurse (other than school nurse)	839 (20.0)	0.3 (0.9)	705 (17.5)	0.3 (0.8)
Hospital appointment	1338 (31.9)	0.6 (1.5)	1200 (29.7)	0.6 (2.1)
Accident & Emergency	827 (19.7)	0.3 (0.7)	737 (18.3)	0.3 (1.1)
Overnight hospital stay	270 (6.5)	0.2 (2.0)	261 (6.5)	0.3 (3.7)
Psychologist	72 (1.7)	0.0 (0.8)	81 (2.0)	0.0 (0.5)
Counsellor (other than at school)	76 (1.8)	0.1 (0.9)	65 (1.6)	0.1 (1.4)
Social worker	142 (3.4)	0.1 (1.7)	112 (2.8)	0.1 (1.6)
Telephone help-line	30 (0.7)	0.0 (0.4)	46 (1.1)	0.0 (0.4)
**Criminal Justice**				
Youth justice service	32 (0.8)	0.0 (0.6)	33 (0.8)	0.0 (0.3)
Police service	108 (2.6)	0.0 (0.4)	120 (3.0)	0.0 (0.4)

Table S4 Public sector service by treatment arm from 18 to 24 months. Values are number (percentages) of pupils using the service and mean (SD) use.

Service	Intervention (n=4189)		Education as normal (n=4037)	
	Number using the service (%)	Mean (SD)	Number using the service (%)	Mean (SD)
**Education**				
School nurse	1403 (33.5)	0.7 (2.2)	1325 (32.8)	0.7 (3.4)
School counsellor/guidance teacher	567 (13.5)	0.4 (3.0)	608 (15.1)	0.5 (3.3)
Educational psychologist	62 (1.5)	0.1 (1.9)	42 (1.0)	0.0 (0.5)
Education welfare officer/ home-school liaison officer	84 (2.0)	0.1 (1.3)	55 (1.4)	0.0 (0.5)
**Health**				
GP surgery visit	2013 (48.1)	0.9 (2.2)	1978 (49.0)	0.9 (1.4)
GP out of hours	498 (11.9)	0.2 (1.8)	462 (11.4)	0.2 (0.6)
Nurse (other than school nurse)	768 (18.3)	0.3 (2.2)	752 (18.6)	0.3 (1.0)
Hospital appointment	1269 (30.3)	0.6 (2.7)	1269 (31.4)	0.6 (0.8)
Accident & Emergency	764 (18.2)	0.3 (2.3)	810 (20.1)	0.3 (0.8)
Overnight hospital stay	245 (5.9)	0.3 (3.3)	266 (6.6)	0.3 (7.3)
Psychologist	86 (2.1)	0.1 (2.6)	91 (2.3)	0.1 (1.0)
Counsellor (other than at school)	111 (2.7)	0.1 (2.8)	104 (2.6)	0.1 (1.1)
Social worker	137 (3.3)	0.6 (3.3)	132 (3.3)	0.1 (1.2)
Telephone help-line	42 (1.0)	0.1 (3.0)	48 (1.2)	0.1 (0.9)
**Criminal Justice**				
Youth justice service	31 (0.7)	0.1 (3.2)	30 (0.7)	0.0 (1.0)
Police service	144 (3.4)	0.1 (3.3)	129 (3.2)	0.1 (1.6)

Table S5 Public sector service by treatment arm from 27 to 33 months. Values are number (percentages) of pupils using the service and mean (SD) use.

**Service**	**Intervention (n=4189)**		**Education as normal (n=4037)**	
	**Number using the service (%)**	**Mean (SD)**	**Number using the service (%)**	**Mean (SD)**
**Education**				
School nurse	1195 (28.5)	0.6 (2.2)	1094 (27.1)	0.5 (1.4)
School counsellor/guidance teacher	558 (13.3)	0.6 (3.7)	560 (13.9)	0.6 (3.8)
Educational psychologist	41 (0.98)	0.0 (0.4)	39 (1.0)	0.0 (0.4)
Education welfare officer/ home-school liaison officer	64 (1.5)	0.1 (4.5)	56 (1.4)	0.0 (0.5)
**Health Sector**				
GP surgery visit	1943 (46.4)	1.0 (1.8)	1790 (44.3)	1.0 (2.2)
GP out of hours	399 (9.5)	0.1 (0.6)	350 (8.7)	0.1 (0.7)
Nurse (other than school nurse)	622 (14.9)	0.3 (0.9)	593 (14.7)	0.3 (1.0)
Hospital appointment	1073 (25.6)	0.6 (1.7)	975 (24.2)	0.5 (2.0)
Accident & Emergency	635 (15.2)	0.2 (0.6)	626 (15.5)	0.2 (1.0)
Overnight hospital stay	169 (4.0)	0.1 (1.0)	161 (4.0)	0.1 (1.0)
Psychologist	85 (2.0)	0.1 (1.5)	73 (1.8)	0.1 (0.8)
Counsellor (other than at school)	96 (2.29)	0.2 (1.6)	98 (2.4)	0.1 (1.6)
Social worker	85 (2.03)	0.1 (1.3)	108 (2.7)	0.1 (1.1)
Telephone help-line	43 (1.0)	0.0 (0.6)	39 (1.0)	0.0 (0.4)
**Criminal Justice Service**				
Youth justice service	10 (0.2)	0.0 (0.3)	18 (0.5)	0.0 (0.3)
Police service	97 (2.3)	0.1 (0.5)	111 (2.8)	0.0 (0.6)

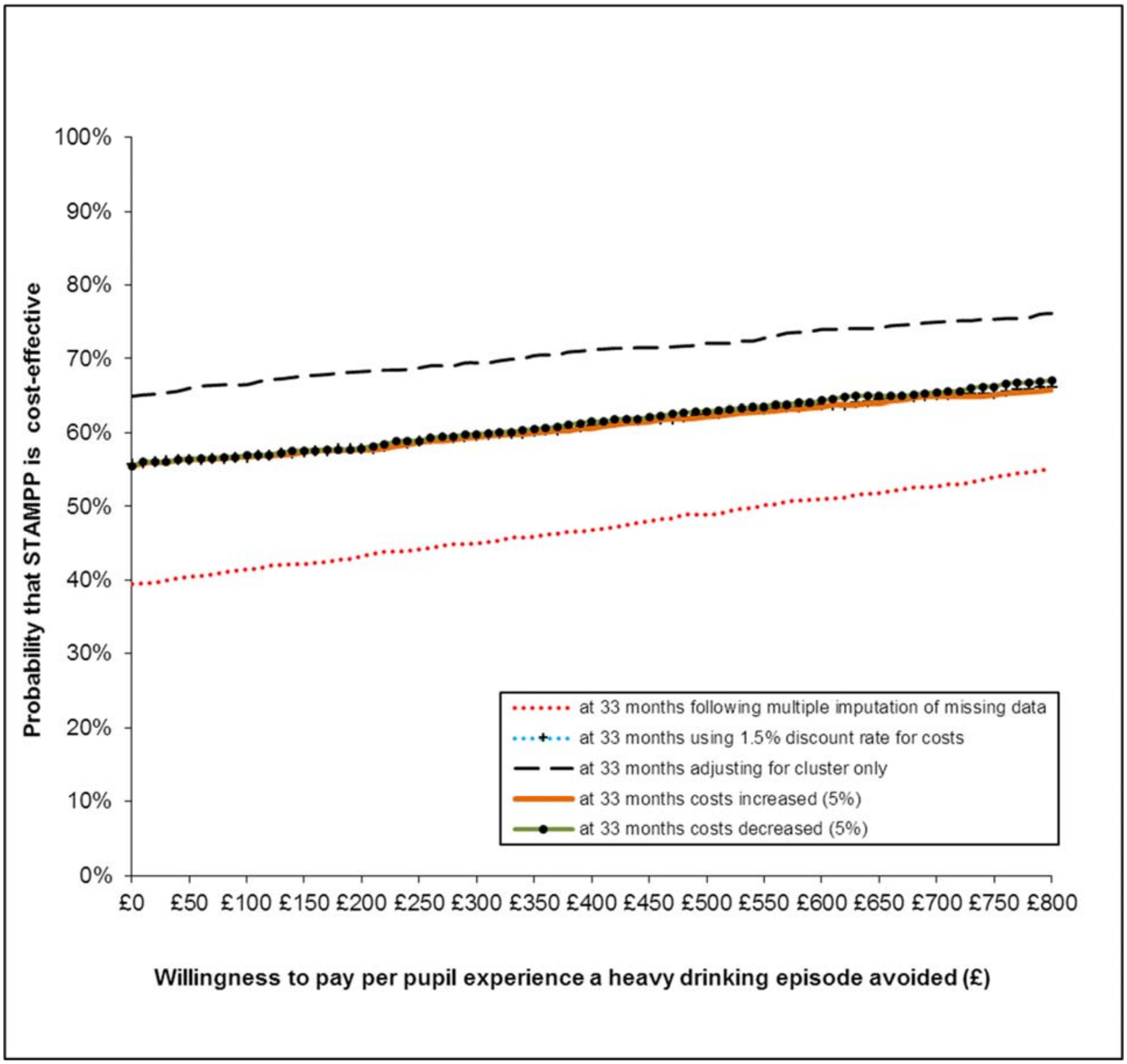

Figure S1 Cost effectiveness acceptability curves showing the probability of STAMPP being cost-effective compared to usual education for the sensitivity analyses.

Table S6 Baseline characteristics of pupils' with complete and non-complete health economic data by intervention arm. Values are counts [percentage] or mean [95% Confidence interval]

Baseline characteristics	Complete data (included in cost-effectiveness analysis)			Incomplete data (excluded from cost-effectiveness analysis)		
	Intervention n=4189 [%]	Education as normal n=4037[%]	Total n=8226 [%]	Intervention n=2190 [%]	Education as normal n=2322[%]	Total n=4512 [%]
School location						
NI	2764 [66]	2637 [65]	5401 [66]	1085 [50]	1256 [54]	2341 [52]
Scotland	1425 [34]	1400 [35]	2825 [34]	1105 [50]	1066 [46]	2171 [48]
*School free school meal category**						
Low	1720 [41]	1442 [36]	3162 [38]	617 [28]	509 [22]	1126 [25]
Moderate	1672 [40]	1469 [36]	3141 [38]	880 [40]	991 [43]	1871 [41]
High	797 [19]	1126 [28]	1923 [23]	693 [32]	822 [35]	1515 [34]
School type						
Co-education	3353 [80]	3229 [80]	6582 [80]	1910 [87]	1989 [86]	3899 [86]
Girls	541 [13]	394 [10]	935 [11]	175 [8]	114 [5]	289 [6]
Boys	295 [7]	414 [10]	709 [9]	105 [5]	219 [9]	324 [7]
Pupil reported heavy episodic drinking						
Yes	198 [5]	213 [5]	411 [5]	233 [11]	219 [9]	452 [10]
No	3588 [86]	3319 [82]	6907 [84]	1673 [76]	1763 [76]	3436 [76]
Missing	403 [10]	505 [13]	908 [11]	284 [13]	340 [15]	624 [14]

* Percentage of n (n=Free school meal provision- Low; 0-15.4%, Moderate; 15.5-30.4%, High; 30.5% and above

Table S7 Public service costs (£UK) in the 6 months pre-baseline (reported at baseline).

Baseline characteristic	Complete data (included in cost-effectiveness analysis)			Incomplete data (excluded from cost-effectiveness analysis)		
	Intervention n=4189	Education as normal n=4037	Total n=8226	Intervention n=2190	Education as normal n=2322	Total n=4512
Mean [95% CI]	620 [481-759]	631 [487-774]	625 [525-725]	667 [572-761]	698 [612-783]	682 [619, 746]

Missing (n; %)	371 [9]	484 [12]	855 [10]	259 [12]	308 13]	567 [13]

Alternative intervention costs

We explored two alternative scenarios for delivering STAMPP- 1) the removal of the parental component making the intervention classroom based only, 2) the removal of the parental; component and the the costs associated with teaching time (to reflect STAMPP being incorporated in to the curriculum).

Table S8 Total cost to deliver STAMPP

**Stage 1 Planning and preparation for delivery**	Total Cost (£)
Materials	£6,694
Training	£38,079
**Stage 1 subtotal**	**£44,773**
**Stage 2 Delivery**	
Teaching	£27,877
Facilitator (for parental component)	£13,250
**Stage 2 subtotal**	**£41,127**
**STAMPP Total Cost**	**£85,900**
Mean cost/school[1]	**£818**
Mean cost/pupil[2]	**£15**
**Classroom component only- Total Cost[3]**	**£72,650**
Mean cost/school[1]	**£692**
Mean cost/pupil[2]	**£13**
**Materials and teacher training only- Total Cost[4]**	**£44,773**
Mean cost/school[1]	**£426**
Mean cost/pupil[2]	**£8**

[1]based on 5749 pupils at baseline and 192 classes

[2]based on 105 schools

[3]excluding parental component cost of £13,250 for facilitator

[4] based on Stage 1 costs only
