# Supplementary material for: Cost-effectiveness of a combined classroom curriculum and parental intervention: economic evaluation of data from the Steps Towards Alcohol Misuse Prevention Programme cluster randomised controlled trial
Source: BMJ Open. 2019 Jul 2;9(7):e027951. doi: 10.1136/bmjopen-2018-027951 (PMC6609141; doi:10.1136/bmjopen-2018-027951)
Supplement: Author's manuscript [file bmjopen-2018-027951.draft_revisions.pdf]

BMJ Open is committed to open peer review. As part of this commitment we make the peer review history of every article we publish publicly available.

When an article is published we post the peer reviewers' comments and the authors' responses online. We also post the versions of the paper that were used during peer review. These are the versions that the peer review comments apply to.

The versions of the paper that follow are the versions that were submitted during the peer review process. They are not the versions of record or the final published versions. They should not be cited or distributed as the published version of this manuscript.

BMJ Open is an open access journal and the full, final, typeset and author-corrected version of record of the manuscript is available on our site with no access controls, subscription charges or pay-per-view fees (http://bmjopen.bmj.com).

If you have any questions on BMJ Open's open peer review process please email
info.bmjopen@bmj.com

BMJ Open

Cost-effectiveness of a combined classroom curriculum and parental intervention: economic evaluation of data from the Steps Towards Alcohol Misuse Prevention Programme (STAMPP) cluster randomised controlled trial.

Journal:	*BMJ Open*
Manuscript ID	bmjopen-2018-027951
Article Type:	Research
Date Submitted by the Author:	15-Nov-2018

Complete List of Authors:	Agus, Ashley; Northern Ireland Clinical Trials Unit, Health Economics<br>McKay, Michael; University of Liverpool School of Life Sciences, Psychological Sciences<br>Cole, Jonathan; University of Liverpool<br>Doherty, Paul; Northern Ireland Clinical Trials Unit<br>Foxcroft, David; Oxford Brookes University UK<br>Harvey, Séamus<br>Murphy, Lynn; Northern Ireland Clinical Trials Unit<br>Percy, Andrew; Queen's University Belfast, Social Sciences, Education and Social Work<br>Sumnall, Harry; Liverpool John Moores University, Public Health Institute

Keywords:	PUBLIC HEALTH, HEALTH ECONOMICS, EDUCATION & TRAINING (see Medical Education & Training), Community child health < PAEDIATRICS, Alcohol misuse, School-based

SCHOLARONE™
Manuscripts

Cost-effectiveness of a combined classroom curriculum and parental intervention: economic evaluation of data from the Steps Towards Alcohol Misuse Prevention Programme (STAMPP) cluster randomised controlled trial.

Corresponding author: Ashley Agus[1]

Email: ashley.agus@nictu.hscni.net

Authors:

Ashley Agus[1], Michael McKay[2], Jon Cole[2], Paul Doherty[1], David R. Foxcroft[3], Séamus Harvey[4], Lynn Murphy[1], Andrew Percy[5], Harry Sumnall[6]

[1] Northern Ireland Clinical Trials Unit, The Royal Hospitals, Belfast, UK

[2] Department of Psychological Sciences, University of Liverpool, UK

[3] Psychology and Public Health, Oxford Brookes University, UK

[4] School of Sport, Health and Exercise Sciences, University of Bangor, UK

[5] School of Social Sciences, Education, and Social Work, Queen's University Belfast, UK

[6] Public Health Institute, Liverpool John Moores University, UK

For peer review only

Abstract (word count 291)

Objectives: To assess the cost-effectiveness of the Steps Towards Alcohol Misuse Prevention Programme (STAMPP) compared with education as normal (EAN) in reducing self-reported heavy episodic drinking in adolescents.

Design: A cost-effectiveness analysis from a public sector perspective conducted as part of a cluster randomised trial

Setting: 105 High schools in Northern Ireland (NI) and in Scotland.

Participants: Students in school year 8/S1 (aged 11-12) at baseline..

Interventions: A classroom-based alcohol education curricula, combined with a brief alcohol intervention for parents/carers.

Outcome measures: Cost per young person experiencing heavy episodic drinking avoided due to STAMPP at 33 months from baseline.

Results The total cost of STAMPP was £85,900, equivalent to £818 per school and £15 per pupil. Due to very low uptake, we calculated costs of £692 per school and £13 per pupil without the parental component. Costs per pupil were reduced further to £426 per school and £8 per pupil when it was assumed there were no additional costs of classroom delivery if STAMPP was delivered as part of activities such as Personal, social, health and economic education (PSHE). STAMPP was associated with a significantly greater proportion of pupils experiencing a heavy drinking episode avoided (0.08/8%) and slightly lower public sector costs (mean difference -£17.19). At a notional willingness-to-pay threshold of £15 (reflecting the cost of STAMPP) the probability of STAMPP being cost-effective was 56%. This level of uncertainty reflected the substantial variability in the cost differences between groups.

Conclusions: STAMPP was relatively low-cost and reduced heavy episodic drinking. STAMPP was not associated with any clear public sector cost-savings, but neither did it increase them or lead to any cost-shifting within the public sector categories. Further research is required to establish if the cost-effectiveness of STAMPP is sustained in the long-term.

Trial registration: ISRCTN47028486. The date of registration was 23/09/2011, and school recruitment began 01/11/2011.

Strengths and limitations of this study

- A broad public sector perspective was adopted for the analysis.
- Participant level service use data was obtained directly from the students thus avoiding the need to involve parents/ guardians in questionnaire completion.
- Study was not specifically powered to detect statistically significant differences in costs or cost-effectiveness.
- Only two-thirds of the students had complete cost and outcome data, the rest we assumed were missing at random.

For peer review only

Introduction

Although prevalence of alcohol use in children and young people is generally decreasing in the United Kingdom (UK), it is high in comparison to many European countries[1-5] . Children and young people who engage in heavy drinking exposure themselves to a wide range of negative physical and mental health, social, legal and educational risks [6-9] (Bonomo, et al 2001; Donoghue et al., 2017; Newbury-Birch et al., 2008; Odgers et al 2008). As alcohol consumption patterns established during adolescence appear to persist well into adulthood [10,11] (Degenhardt et al., 20013; Percy and McKay 2015), heavy drinking in adolescence may contribute to increased health and social services utilisation, placing a substantial economic burden on society [12,13] (James & Francesconi, 2015; Levy, Miller and Cox 1999).. Indeed, considering the external costs imposed on society only, the annual burden of alcohol for England and Wales, Scotland, and Northern Ireland are reportedly £21 billion, £2.1 billion and £680 million respectively [14]. Heavy episodic (binge) drinking costs alone have been estimated at £4.86 billion per year (£77 per capita at 2014 prices)[12]. Given the complexity and scale of the problem, multiple system-wide interventions are required to address the burden of alcohol-related harm, including cost-effective prevention interventions to reduce binge drinking in young people.

In addition to environmental and policy led approaches to alcohol (e.g. pricing and marketing restrictions) [15], systematic reviews suggest that some universal alcohol prevention interventions are effective in reducing use in young people [16]. Although existing (2007) National Institute for Health and Care Excellence (NICE) guidelines on school-based alcohol prevention (updated expected 2019) do not recommend any single prevention programme, they called for partnership working between schools and other stakeholders in efforts to prevent use [17]. NICE suggested that school-based interventions should aim to increase knowledge about alcohol, to explore perceptions about alcohol use and to help develop decision-making skills, self-efficacy and self-esteem. The guideline also highlighted the paucity of evidence from economic evaluations of school-based programmes, although a few additional studies have subsequently emerged (see Hill et al., 2017 for a recent review; 18).

The Steps Towards Alcohol Misuse Prevention Programme (STAMPP) cluster randomised controlled trial was undertaken to assess the effectiveness of a combined school-based universal alcohol harm reduction curriculum and a brief parental intervention [19, 20, 21]. The

For peer review only

trial found that the STAMPP intervention was well received by both pupils and school staff and it reduced self-reported heavy episodic drinking (HED) in the past 30 days at 33 month follow-up from baseline, compared with education as normal (EAN). However, compliance with the parental component was low, and the intervention did not reduce alcohol-related harms associated with own drinking which were low in both trial arms, but it was concluded that effects on harms might manifest later.

The aim of this paper is to report on the cost-effectiveness of STAMPP compared with EAN in reducing self-reported heavy episodic drinking in adolescents.

For peer review only

Methods

The STAMPP trial has described in detail elsewhere [19, 20]. It recruited 12,738 participants (Intervention=6379, Education as Normal=6359) from 105 schools in Northern Ireland and the Glasgow and Inverclyde areas of Scotland. All participants were in year 8/S1 (aged 11-12) at baseline (June 2012). We performed an incremental cost-effectiveness analysis alongside the trial to estimate the cost per young person experiencing heavy episodic drinking avoided due to STAMPP at 33 months. A public sector perspective was adopted for the analysis, which encompassed the costs to Local Authorities, National Health Service (NHS), Personal Social Services (PSS) and Criminal Justice Service.

Outcomes

Consistent with the primary outcome of the study, the primary economic effectiveness measure was the number of pupils who reported any HED in the previous 30 days at 33 months. This was based on responses to the question *"How often in the past month have you drank 4.5 (female) / 6 (male) or more units of alcohol?"* Descriptive statistics were used to summarise the proportion of pupils in each arm reporting a heavy drinking episode.

Costs

Intervention resources use

We calculated the economic cost of STAMPP according to the principle of opportunity cost, that is, we attempted to place a value on the benefits which were foregone by STAMPP being delivered instead of something else. We therefore included the full value of all the resources it used, regardless of whether the resources were directly purchased for the study. The process

evaluation carried out alongside the trial established that STAMPP was delivered in most schools as part of their personal, social, health and economic (PSHE) (or local equivalent) provision and that curriculum-based alcohol education activities were found to be minimal in both control and intervention schools [19]. We therefore made the very conservative assumption that there were no costs associated with EAN, that is, STAMPP was seen as an additional cost to EAN and the costs of other alcohol education activities were not included in the analysis.

Resources were categorised according to the stage they were used in the research process; planning and preparation for delivery (stage 1), and delivery itself (stage 2), in keeping with other trials of behavioural intervention [22-24]. Pre-start-up resources associated with the development of STAMPP were not included in the analyses as they would not be incurred should the intervention be mainstreamed in the future. These included the development of the teacher manual and pupil work book content, planning the lessons and the design of materials.

Stage 1 resources in STAMPP covered; recurring costs associated with printing/copying the intervention materials (teacher manual, pupil work book, parent/guardian information sheets, CD-ROMs) and the delivery of teacher training (trainer fees, venue, catering and teacher cover). No costs associated with the purchasing and licensing of the STAMPP intervention are foreseen and so are not included here. Stage 2 resources covered recurring costs associated with teacher time input for delivery of Phase 1 and Phase 2 of the programme. The cost of teaching time was included, even though the intervention was delivered within normal working hours and so would not have been an extra cost to the public sector. In doing so we captured the opportunity cost of the benefits to pupils which were foregone by STAMPP being delivered instead of other lessons.

Resources used during stages 1 and 2 were recorded retrospectively by the project team and costs were obtained from invoices when available. The costs associated with printing the intervention materials were not available as these resources were present at the start of the trial. We therefore obtained printing quotations and deflated it to the appropriate price year using the Consumer Price Index [25]. The Local Authority in Glasgow/Inverclyde covered all the costs associated with the training of the teachers including teacher cover, venue and catering and so these were also not available. We therefore used the costs that were available for NI and

For peer review only

applied it to Scotland. The majority of teachers delivering the intervention were at a junior level, thus we used point 3 on the Main Pay Scale [26] including 24% for employers' costs.

Stage 1 costs were added together to get a total cost. Stage 2 costs relating to the delivery of the intervention at a class level were multiplied by the total number of classes that received intervention to get a total cost. This was estimated by dividing the number of pupils receiving the intervention at baseline (n=5749) by 30, which was used as an estimate of the maximum number of pupils per class. Stage 2 costs relating to the delivery of the parental component were calculated by multiplying the number of schools by the costs to deliver one session. Stage 1 and Stage 2 costs were totalled and divided by the number of pupils receiving the intervention at baseline to obtain the mean cost per pupil.

Students' service use

Data on service use by all pupils from baseline to 33 months was collected using an instrument administered at baseline, 12 months, 24 months and 33 months [19]. The instrument incorporated some items taken from the Client Service Receipt Inventory [27] specifically adapted for childhood [28] and items relating to the use of judicial services. An information page was provided containing definitions of some of the public services in case the students were unfamiliar with them. The instrument was designed with input from relevant professionals (e.g. educational psychologist, social workers, Scottish and NI teachers) and reviewed by a social researcher experienced in delivering questionnaires to children, and other health economists. The instrument asked pupils to report their use of services in the previous six months thus providing service use data for; the 6 months pre-baseline, 7-12 months, 19-24 months and 28-33 months. If there were any missing fields within the service use questionnaire, it was assumed that the relevant service had not been used.

Individual-level service use was combined with unit costs (Table 1) to estimate a cost for each pupil for each of the four survey time periods. Unit costs were obtained from publicly available sources [29-31] and set at 2013-14. For school counsellors/guidance teacher we assumed a mid-point of on the Upper Pay Scale (UPS2)[26] and included 24% for employers' costs. As there were gaps in the survey period where no cost data were collected (baseline to 6 months, 12-18 months and 24-27 months), we used interpolation of the available cost data to fill in these gaps. This was done separately for each participant to obtain an estimate of their total service use costs at 33 months. Total costs could not be calculated for those pupils who had any missing

For peer review only

cost data points as these were required for the interpolation. Descriptive statistics were used to summarise service use and costs at each time point for the different categories of the public sector (Education, Health and Criminal Justice). Costs of service use in the 6 months before baseline were not included in total costs but were used in the interpolation.

[Table 1]

Cost-effectiveness analysis

Cost-effectiveness was estimated using conventional decision rules [32] and reported as incremental cost-effectiveness ratios (ICER) if appropriate. The ICER is a measure of the additional cost per additional unit of effect produced by one intervention compared with another. For an intervention to be considered cost-effective, the ICER must be less than the maximum amount of money that a decision-maker would be willing to pay per unit of effect, that is, their willingness-to-pay (WTP) threshold. A cost-effectiveness analysis (CEA) estimated the incremental cost per young person experiencing HED avoided due to STAMPP at 33 months. To maintain the correlation structure of the data, only pupils with complete cost and outcome data were included in the analyses. This meant pupils with cost data from baseline to 33 months and a response to the primary outcome variable at 33 months.

Bootstrapped multilevel mixed-effects regression models were used to estimate mean incremental costs and effects (with 95% confidence intervals based on 1000 bootstrap resamples). Both models adjusted for school location (NI/ Scotland), school level of FSM provision (Low; 0-15.4%, Moderate; 15.5-30.4%, High; 30.5% and above), school type (all boys' school/ all girls' school/ co-education school) and clustering. In addition, the cost model adjusted for pupil's baseline costs and the effects model adjusted for pupils' baseline drinking. Uncertainty in the cost-effectiveness measures was investigated by using the 1000 resampled incremental costs and effects to generate 1000 replications of the ICERs. The replicates were plotted on the cost-effectiveness plane and used to construct cost-effectiveness acceptability curves (CEACs). The CEACs showed the probability of STAMPP being cost-effective compared with EAN at different threshold levels of WTP to avoid a pupil experiencing an episode of heavy drinking in the previous 30 days at 33 months. As there is no generally accepted threshold value for cost per pupil experiencing any HED avoided we looked at range of thresholds, including the cost of the intervention per pupil, and compared our findings with those of other economic evaluations which have been performed in this research area.

For peer review only

All analyses were performed using Stata 12/IC for Windows®. Costs occurring in the second and third year of the study were discounted at 3.5% in keeping with NICE (2013) guidance [33].

Sensitivity analyses

A number of one-way sensitivity analyses were performed to test the robustness of the cost-effectiveness findings;

- Missing total costs and outcomes were filled simultaneously using multiple imputation by chained equations. In the multiple imputation model we included all of the variables which were to be included in the subsequent multilevel models i.e. the treatment variable, baseline costs, baseline drinking, school location, school level of entitlement to free meals and clustering. We also included 6-12 month costs and 6-12 month drinking. We used predictive mean matching for costs and a logit model for the primary outcome. Five imputed data sets were generated and the results combined.
- Total costs were discounted at a rate of 1.5% as suggested by NICE [34] for public health interventions.
- The multilevel models were re-estimated without adjusting for baseline covariates but still adjusting for clustering.
- Since a linear time trend was assumed between data time points this might have led to total costs and total heavy drinking episodes being under/over-estimated if said trend is not appropriate. The impact of increasing and decreasing total costs by 5% were therefore explored.

Results

There were 11,316 pupils present at the baseline assessment; 5749 in the intervention group and 5567 in EAN. A total number of 12738 pupils took part in the study, with 6379 in the intervention group and 6359 in usual education. Approximately two-thirds of all pupils had complete cost and outcome data and were included in the analysis. This was similar across groups (Intervention =66%; 4189/6379) (EAN=63%; 4037/6359).

For peer review only

The resources and costs used in the planning and preparation, and delivery of the intervention are presented in the online supplementary file Table S1. Total costs are presented in Table 2. The mean cost per school was £818 and the mean cost per pupil was £15. The largest proportion of the costs was associated with the training of the teachers as this involved teaching cover, location costs and facilitator costs. The second largest cost was associated with the delivery of the intervention in the classroom setting. In light of the low uptake of the parental component of STAMPP we also present the cost of delivering the STAMPP classroom component only. This reduced costs to £692 per school and £13 per pupil. The cost of the intervention with no parental component and no cost associated with delivering the classroom component (e.g. where trained teachers deliver the intervention as part of PSHE) was £426 per school and £8 per pupil.

[Table 2]

The use of public sector services by all pupils with available data in the 6 months pre-baseline, from 6 to 12 months, from 18-24 months and from 27 to 33 months are presented in the online supplementary file (**Error! Reference source not found.**-S4). For those pupils with complete data, the costs of public services used over the study period following interpolation of costs and adjustment for baseline covariates and clustering are shown in Table 3. Negative costs reflect a cost saving in favour of the intervention. The difference in total public sector costs was small and not statistically significant.

[Table 3]

The proportion of pupils reporting a heavy drinking episode in the previous 30 days is shown in Table 4. The outcomes are reported in terms of cases avoided; thus, a positive difference reflects a smaller number of pupils in the intervention arm experiencing a heavy drinking episode in the previous 30 days. Statistically significantly fewer pupils in the intervention arm reported drinking heavily in the previous 30 days (15 % vs. 23%) compared to control.

[Table 4]

Cost-effectiveness analysis

For peer review only

The results from the primary CEA are shown in Table 5. The cost per pupil for the intervention (£15) was added to each pupil in the intervention arm. STAMPP was associated with a statistically significantly greater proportion of pupils experiencing a heavy drinking episode *avoided* (0.08/8%; 0.06, 0.09) and lower mean total costs (-£17.19; -402.84, 368.46). The difference in costs was small and not statistically significant, therefore STAMPP could be considered cost-neutral. When an ICER is negative, as in this case (-17.19/0.08), its magnitude does not convey any meaning so they are not calculated [36]. STAMPP can be said to dominate usual education; however, since the difference in costs was not statistically different, only weak dominance can be claimed [37]. Uncertainty surrounding the estimates of total costs and outcomes is represented by the bootstrapped ICERs on the cost-effectiveness plane (**Error! Reference source not found.** 1). The majority of points straddle both the north east and south east quadrant indicating that, although STAMPP is likely to be more effective than the usual education, there is considerable variability about the cost estimates. The corresponding CEAC is in Figure 2; when WTP thresholds ranging from £0 to £800 are considered, it can be seen that the probability of STAMPP being cost-effective compared to usual education ranges from 55% to 67%. Uncertainty in the cost-effectiveness of the intervention remains substantial until much higher WTP values, with an 80% probability being displayed at a WTP of £2,000. If decision-makers were only willing to pay the £15 (the cost of STAMPP per pupil) the probability would be 56%.

The results of the sensitivity analyses for the primary CEA are presented in Table 5 and the corresponding CEACs are in the online supplementary file (Figure S1). After the multiple imputation of missing data, the probability of STAMPP being cost-effective was lower at each WTP threshold, ranging from 40-55%. In contrast, when the multilevel models were re-estimated adjusting only for baseline covariates the probability of cost-effectiveness was consistently higher, ranging from 65%-76%. Reducing the discount rate to 1.5% and increasing/decreasing costs by 5% had little effect on the cost-effectiveness of STAMPP.

[Table 5]

Discussion

The total cost to deliver STAMPP was £85,900, equivalent to £818 per school and £15 per pupil. As there was low uptake of the parental component, we calculated costs of £692 per school and £13 per pupil without delivery of the parental component. Costs per pupil were reduced further to £426 per school and £8 per pupil when it was assumed there were no costs

For peer review only

of classroom delivery, as might be the case when previously trained teachers deliver the intervention as part of regular PSHE curriculum (i.e. the classroom component would replace scheduled lessons). In a review of economic evidence for the development of the NICE guidance [17] the cost of other effective school-based interventions range from £20- £150 (cost year 2005/6) [37-39] one of which was Australian School Health and Alcohol Harm Reduction Project (SHAHRP) from which the classroom component of STAMPP was adapted [37]. Another review of school-based skills development substance use prevention curricula in the USA (which targeted both alcohol and illicit drug estimated programme unit costs between US $100-400 per pupil [40]). Thus, at a cost of £15 per pupil, STAMPP is a relatively low-cost intervention that successfully reduces problematic drinking. If costs were extrapolated to the 685,300 students aged 12 years old in the UK in 2013/14 the cost would be £10.3 million in total. This is a relatively small amount compared with the overall annual economic burden from alcohol consumption in the UK.

The analysis of public service costs showed there was an overall reduction in the use of public sector services over the 33 month study period for both groups. There was however no difference between groups, and no differences in the use of the sub-categories of education, health and criminal justice services. Costs were estimated from a public sector perspective which was justified considering one of the principal objectives of STAMPP was to reduce alcohol-related harms in teenagers. It was hypothesised that this would in turn reduce the use of health and judicial services and the need for additional support within the school setting to address behavioural and emotional problems. The absence of a statistically significant difference in public service costs between groups is in keeping with the analysis of the harms data from the trial. No differences were observed in the number of self-reported harms by pupils between groups and indeed both groups reported low levels of harms overall.

Compared to EAN, significantly fewer pupils in the intervention group reported experiencing a heavy drinking episode in the previous 30 days at 33 months. The CEA indicated that STAMPP weakly dominated EAN. At a notional WTP threshold of £15 (reflecting the cost of STAMPP) the probability of STAMPP being cost-effective was 56%. This level of uncertainty reflects the considerable variability in the cost differences between groups. In light of the literature linking excess and early initiation of drinking in adolescence to alcohol use behaviours in adulthood (e.g., the development of poor health outcomes over a sustained period of time [41];, it is important to investigate if the (cost) effectiveness of STAMPP is maintained or even increases in the long-term.

For peer review only

The sensitivity analyses indicated that the results of the CEA were robust to small changes in parameters i.e. discounting and small increases in cost and effectiveness. However, when costs and effects were not adjusted for baseline covariates, the probability of cost-effectiveness of STAMPP increased. This suggests that the cost-effectiveness of STAMPP may vary between sub-groups and warrants further investigation to identify which pupils or schools might benefit the most from receiving the intervention. Furthermore, when multiple imputation was used to impute missing cost and outcome data the cost-effectiveness of STAMPP decreased. Since multiple imputation is based on the assumption that data is missing at random, it may not be appropriate for this data.

The strengths of the economic evaluation include the choice of perspective; we adopted a public sector perspective in keeping with NICE guidance for public health interventions [34] thus we looked beyond the healthcare system and considered the broader impact of the intervention. In contrast, Hill et al (2017)[18] found that the majority of economic evaluations of alcohol prevention considered only health care costs; the danger being that interventions may be undervalued if cost-savings occur in other sectors or overvalued if costs are incurred in other sectors. A further strength was that we obtained resource use data directly from the pupils thus avoiding the need to involve parents/guardians in questionnaire completion. We provided definitions of the services using age appropriate terminology, with input from relevant professionals. Considering the difficulty associated with engaging parents/guardians in the study, reflected in the poor attendance to the parental evenings [19], it is likely that a reliance on parents/guardians would have led to considerable amounts of missing data.

There were also a number of limitations to the economic evaluation. We made the conservative assumption that STAMPP was delivered in addition to EAN when in reality it is likely that STAMMP will have replaced whatever statutory provision was in place, however minimal. By doing this we may have overestimated the cost of STAMPP and not measured what was forgone in order to deliver the intervention. Indeed, in a recent cluster trial of a school-based sexual education intervention [24], teachers indicated that the intervention saved them time which would otherwise have been spent preparing for lessons within the same curriculum module.

For peer review only

The study was not specifically powered to detect statistically significant differences in costs or cost-effectiveness. Although CEA does not typically make decisions based on significance rules [42] having a sufficiently powered study will allow decision-makers to be more confident in the value claim [35]. The resources used during the planning, preparation and delivery of the intervention were largely recorded retrospectively and costs were obtained from invoices when these were available. We endeavoured to use plausible assumptions when actual data were not available, but the consistent and prospective collection of resource use and costs would be lead to more robust data. We included pupils in the CEA only if they had complete cost and effect data. As a result, only two-thirds of the pupils were included in the analysis, the rest we assumed were missing at random. As discussed earlier, this assumption may be flawed. Finally, we did not undertake a formal analysis of the impact of STAMPP on health inequalities, but an equity impact analysis [43] that disaggregates costs and outcomes by equity-relevant sub groups such as gender, receipt of free school meals, school area deprivation, and ethnicity may help us to understand this impact better.

Conclusions

STAMPP was a relatively low-cost intervention that successfully reduced HED. STAMPP did not bring about clear public sector cost-savings; however, neither did it increase them or lead to any cost-shifting within the public sector categories. STAMPP can therefore be considered to weakly dominate EAN because it was both cost-neutral and more effective. Further research is required to establish if the cost-effectiveness of STAMPP is sustained in the long-term.

Acknowledgements

As well as acknowledging the role played by participating schools and school children, the authors would like to acknowledge the support of the following people in this project: Séamus Mullin, Gerry Bleakney, Owen O'Neill (PHANI); Malachy Crudden (CCMS), Maura Kearney, and Fergal Doherty (Psychological Services, Glasgow); Kate Watson (Psychological Services, Inverclyde) and John Butcher and Sandy Cunningham (Education Services, Glasgow). Thank you also to Rita Faria (Centre for Health Economics, University of York) for her valuable feedback on an early draft of the manuscript. The views and opinions expressed therein are those of the authors and do not necessarily reflect those of the NIHR-PHR, NIHR, NHS or the Department of Health.

For peer review only

Contributions: Sumnall had full access to all of the data in the study and takes responsibility for the integrity of the data and the accuracy of the data analysis. Agus undertook the health economic analysis, wrote the first draft of the manuscript and subsequent versions, and submitted the final version; Sumnall was project PI, contributed to the first draft and subsequent iterations of the manuscript; Cole, Doherty, Foxcroft Harvey, McKay, Murphy, Percy all contributed to drafts and approved the submission.

Funding: This trial was funded by the National Institute of Health Research (NIHR) Public Health Research (PHR) programme (project number 10/3002/09). The Public Health Agency of NI and Education Boards of Glasgow/Inverclyde provided some intervention costs. Diageo provided funds to print classroom workbooks for use only in the Glasgow Local Authority area. Remaining intervention costs were internally funded.

Disclaimer: The views and opinions expressed therein are those of the authors and do not necessarily reflect those of the NIHR-PHR, NIHR, NHS or the Department of Health. The research and intervention funders had no involvement in intervention design; design and conduct of the study; collection, management, analysis, and interpretation of the data; and preparation, review or approval of the manuscript.

List of abbreviations

CEA -Cost-effectiveness analysis

CEAC - Cost-effectiveness acceptability curves

EAN- Education as normal

HED- Heavy episodic drinking

ICER- Incremental cost-effectiveness ratios

NICE- National Institute for Health and Care Excellence

NHS- National Health Service

NI- Northern Ireland

PSS- Personal Social Services

PSHE - Personal, social, health and economic education

STAMPP- Steps Towards Alcohol Misuse Prevention Programme

SHAHRP- Australian School Health and Alcohol Harm Reduction Project

UK - United Kingdom

WTP - Willingness-to-pay

For peer review only

Ethics approval

The research was approved by Liverpool John Moores University Research Ethics Committee (11/HEA/097). Consent was obtained from school head-teachers/principals before randomisation. Consent from participants and their parents/guardians was obtained after randomisation.

Participant consent: obtained

Competing interests: No personal competing interests declared. The sponsor University (LJMU) received and administered a payment from the alcohol industry for printing of student workbooks in the Glasgow trial site only. Percy reported that he has previously received funding from the European Foundation of Alcohol Research (ERAB) in relation to the development of statistical models for longitudinal data (2008-2010). Foxcroft reported that his Department has previously received funding from the alcohol industry for unrelated prevention programme training work. Sumnall reported that his Department has previously received funding from the alcohol industry (indirectly via the industry funded Drinkaware charity) for unrelated primary research.

Data: The datasets generated during and/or analysed during the current study are not yet publicly available due to the authors undertaking additional analyses and follow-on studies, but are available from the corresponding author on reasonable request.

References

1. NHS Digital (2016) Smoking, drinking and drug use among young people. Available from https://files.digital.nhs.uk/07/49FE46/sdd-2016-rep-cor.pdf
2. Scottish Government (2016) Scottish Schools Adolescent Lifestyle and Substance Use Survey (SALSUS): Alcohol Report 2015. Available from https://beta.gov.scot/publications/scottish-schools-adolescent-lifestyle-substance-use-survey-salsus-alcohol-report/pages/3/
3. Northern Ireland Department of health (2017) Public health NI fact sheet 2017. Available from https://www.health-ni.gov.uk/publications/public-health-ni-fact-sheet-2017 5.
4. ESPAD Group (2016). ESPAD Report 2015. Results from the European School Survey Project on Alcohol and Other Drugs. Luxembourg, Publications Office of the European Union.
5. World Health Organization (2018) Adolescent alcohol-related behaviours: trends and inequalities in the WHO European Region, 2002–2014. Geneva: World Health Organization.

For peer review only

6. Bonomo, Y., Coffey, C., Wolfe, R., Lynskey, M., Bowes, G., & Patton, G. (2001). Adverse outcomes of alcohol use in adolescents. Addiction, 96(10), 1485-1496.
7. Donoghue, K., Rose, H., Boniface, S., Deluca, P., Coulton, S., Alam, M. F., & McArdle, P. (2017). Alcohol consumption, early-onset drinking, and health-related consequences in adolescents presenting at emergency departments in England. Journal of Adolescent Health, 60(4), 438-446.
8. Newbury-Birch D, Gilvarru E, McArdle P, Ramesh V, Stewart S, Walker JB, et al. Impact of Alcohol Consumption on Young People: A Review of Reviews. London: Department for Children, Schools and Families; 2008.
9. Odgers, C. L., Caspi, A., Nagin, D. S., Piquero, A. R., Slutske, W. S., Milne, B. J.,& Moffitt, T. E. (2008). Is it important to prevent early exposure to drugs and alcohol among adolescents?. Psychological Science, 19(10), 1037-1044.
10. Degenhardt, L., O'Loughlin, C., Swift, W., Romaniuk, H., Carlin, J., Coffey, C., & Patton, G. (2013). The persistence of adolescent binge drinking into adulthood: findings from a 15-year prospective cohort study. BMJ open, 3(8), e003015.
11. Percy, A., & McKay, M. (2015). The stability of alcohol consumption between age 16 and 26: Evidence from a National Birth Cohort Study. Journal of Adolescence, 44, 57-69.
12. James, J., & Francesconi, M. (2015). IPR Policy Brief - The cost of binge drinking in the UK. University of Bath.
13. Levy DT, Miller TR, Cox KC. (1999). Costs of Underage Drinking. Washington, DC: US Department of Justice, Office of Justice Programs, Office of Juvenile Justice and Delinquency Prevention
14. Institute of Alcohol Studies. The economic impacts of alcohol. June 2016. Available at: http://www.ias.org.uk/Alcohol-knowledge-centre/Economic-impacts/Factsheets/Estimates-of-the-cost-of-alcohol.aspx (last accessed 31/08/18)
15. Burton, R., et al. (2017). "A rapid evidence review of the effectiveness and cost-effectiveness of alcohol control policies: an English perspective." The Lancet 389(10078): 1558-1580.
16. Foxcroft, D. R. and A. Tsertsvadze (2012). "Universal alcohol misuse prevention programmes for children and adolescents: Cochrane systematic reviews." Perspect Public Health 132(3): 128-134.
17. National Institute for Health and Care Excellence (NICE) (2007) Alcohol: school-based interventions. London: NICE; 2007.
18. Hill, S.R., Vale, L., Hunter, D., Henderson, E., Oluboyede, Y., 2017. Economic evaluations of alcohol prevention interventions: Is the evidence sufficient? A review of methodological challenges. Health policy 121(12), 1249-1262.
19. Sumnall H, Agus A, Cole J, Doherty P, Foxcroft D, Harvey S, et al. Steps Towards Alcohol Misuse Prevention Programme (STAMPP): a school- and community-based cluster randomised controlled trial. Public Health Res 2017;5(2).
20. McKay M, Agus A, Cole J, Doherty P, Foxcroft D, Harvey S, Murphy L, Percy A, Sumnall H. Steps Towards Alcohol Misuse Prevention Programme (STAMPP): a school-based and community-based cluster randomised controlled trial. BMJ Open. 2018 Mar 9;8(3):e019722. doi: 10.1136/bmjopen-2017-019722

For peer review only

21. Harvey, S. A., et al. (2016). "Adolescents' reflections on school-based alcohol education in the United Kingdom: education as usual compared with a structured harm reduction intervention." Journal of Substance Use 21(6): 640-645.
22. Hollingworth W, Cohen D, Hawkins J, Hughes RA, Moore LA, Holliday JC, Audrey S, Starkey F, Campbell R. Reducing smoking in adolescents: cost-effectiveness results from the cluster randomized ASSIST (A Stop Smoking In Schools Trial).Nicotine Tob Res. 2012 Feb;14(2):161-8. doi: 10.1093/ntr/ntr155.
23. Mytton J, Ingram J, Manns S, Stevens T, Mulvaney C, Blair P, Powell J, Potter B, Towner E, Emond A, Deave T, Thomas J, Kendrick D, Stewart-Brown S. The feasibility of using a parenting programme for the prevention of unintentional home injuries in the under-fives: a cluster randomised controlled trial. Health Technol Assess. 2014 Jan;18(3):1-184. doi: 10.3310/hta18030
24. Lohan M, Aventin Á, Maguire L, Curran R, McDowell C, Agus A, et al. Increasing boys' and girls' intentions to avoid teenage pregnancy: a cluster randomised controlled feasibility trial of an interactive video drama-based intervention in post-primary schools in Northern Ireland. Public Health Res 2017;5(1).
25. Office for National Statistics. (2015). CPI annual percentage change: 1989 to 2015. Retrieved from http://www.ons.gov.uk/ons/datasets-and-tables/data-selector.html?cdid=D7G7&dataset=mm23&table-id=1.2 (accessed Dec 9 2015)
26. Department of Education Northern Ireland. Circular 2014/13 - Teachers' pay and allowances from 1 September 2013. 2014. Available from https://www.deni.gov.uk/publications/circular-201413-teachers-pay-and-allowances-1-september-2013 (accessed Dec 9 2015)
27. Beecham J, Knapp M. Costing psychiatric interventions. In Measuring Mental Health Needs (eds G. Thornicroft, C.R.Brewin & J.Wing), pp.163-183. London:Gaskell; 1992.
28. Knapp M, Scott S, Davies J. The cost of antisocial behaviour in younger children. Clinical Child Psychology and Psychiatry. 1999; 4: 457-473.
29. Department of Health. NHS Reference Costs 2013-14, Department of Health, London. 2014.
30. Curtis L, ed. Unit Costs of Health and Social Care 2014. Canterbury: University of Kent: Personal Social Services Research Unit. 2014.
31. National Society for Prevention of Cruelty to Children. Piece by Piece. Annual report and account 2014/15. 2015. Available from https://www.nspcc.org.uk/globalassets/documents/annual-reports/nspcc-annual-report-2014-15.pdf (accessed Dec 9 2015)
32. Johannesson M, Weinstein S. On the decision rules of cost- effectiveness analysis. J Health Econ 1993;12:459–67
33. National Institute for Health and Care Excellence. Guide to the methods of technology appraisal 2013. NICE: London. 2013.
34. National Institute for Health and Care Excellence. Methods for the Development of NICE Public Health Guidance. London: NICE; 2012.
35. Glick HA, Doshi JA, Sonnad SS, Polsky D. Economic evaluation in clinical trials. Oxford: Oxford University Press 2007

For peer review only

36. Drummond M, Sculpher M, Torrance G, O'Brien B, Stoddart G. (2005). Methods for the Economic Evaluation of Health Care Programmes. Third Edition. OUP. Oxford.
37. McBride N, Farringdon F, Midford R. et al. (2004). Harm minimization in school drug education: final results of the School Health and Alcohol Harm Reduction Project (SHAHRP). Addiction 99 (3): 278-291.
38. Eisen M, Zellman GL, and Murray DM. (2003). Evaluating the Lion's-Quest "Skills for Adolescence" drug education program. Second-year behavior outcomes.Addictive Behaviors 28 (5): 883-897.
39. Werch CE., Pappas DM., Carlson JM. et al. (2000b). Evaluation of a brief alcohol prevention program for urban school youth. American Journal of Health Behavior 24 (2): 120-131.
40. Miller, T.R., Hendrie, D., 2008. Substance Abuse Prevention Dollars and Cents: A Cost-Benefit Analysis. Center for Substance Abuse Prevention, Substance Abuse and Mental Health Services Administration.
41. Rehm, J., Baliunas, D., Borges, G.L.G., Graham, K., Irving, H., Kehoe, T., Parry, C.D., Patra, J., Popova, S., Poznyak, V., Roerecke, M., Room, R., Samokhvalov, A.V., Taylor, B., 2010. The relation between different dimensions of alcohol consumption and burden of disease: an overview. Addiction 105(5), 817-843.
42. Claxton K. The irrelevance of inference: a decision-making approach to the stochastic evaluation of health care technologies. J Health Econ. 1999 Jun;18(3):341-64.
43. Cookson, R., Mirelman, A.J., Griffin, S., Asaria, M., Dawkins, B., Norheim, O.F., Verguet, S., Culyer, A.J., 2017. Using Cost-Effectiveness Analysis to Address Health Equity Concerns. Value in Health 20(2), 206-212.

For peer review only

Table 1 Unit costs (UK £) of public sector services

Service	Unit Cost	Source
**Education**		
School nurse	50.00	Unit Costs of Health and Social Care 2014 [30] p.85
School counsellor/guidance teacher	35.02	Department of Education Northern Ireland [26] (per 1 hour of teacher time, point 2 of upper pay scale)
Intervention teacher	25.89	Department of Education Northern Ireland [26] (per 1 hour of teacher time, point 3 of main pay scale)
Educational psychologist	41.00	Unit Costs of Health and Social Care 2014 [30] p.156
Education welfare officer/ home-school liaison officer	27.00	Unit Costs of Health and Social Care 2014 [30] p.155
**Health**		
GP surgery visit	46.00	Unit Costs of Health and Social Care 2014 [30] p.195
GP out of hours	115.00	Unit Costs of Health and Social Care 2014 [30] p.191 (home visit unit cost assumed as above)
Nurse (other than school nurse)	13.70	Unit Costs of Health and Social Care 2014 [30] p.192 (per 15.5 minute surgery consultation)
Hospital appointment	109.00	Unit Costs of Health and Social Care 2014 [30] p.111
Accident & Emergency	233.00	Unit Costs of Health and Social Care 2014 [30] p.111 (see and treat and convey)
Overnight hospital stay	658.33	NHS Reference Costs 2013-14 [29] (weighted average length of stay and cost of paediatric non-elective long stays)
Psychologist	50.00	Unit Costs of Health and Social Care 2014 [30] p.51
Counsellor (other than at school)	50.00	Unit Costs of Health and Social Care 2014 [30] p.51
Social worker	79.00	Unit Costs of Health and Social Care 2014 [30] p.206 (per 1 hour including travel)
Telephone help-line	3.99	National Society for the Prevention of Cruelty to Children Annual Report and Account 2014/2015 [REF] cost per call to Childline deflated to 2013–14
**Criminal Justice**		
Youth justice service	84.00	Unit Costs of Health and Social Care 2014 [REF31] p.224 (face-to-face contact)
Police service	325.00	Unit Costs of Health and Social Care 2014 [30] p.149

For peer review only

Table 2 Total cost to deliver STAMPP

**Stage 1 Planning and preparation for delivery**	Total Cost (£)
Materials	£6,694
Training	£38,079
**Stage 1 subtotal**	**£44,773**
**Stage 2 Delivery**	
Teaching	£27,877
Facilitator	£13,250
**Stage 2 subtotal**	**£41,127**
**STAMPP Total Cost**	**£85,900**
Mean cost/school[1]	**£818**
Mean cost/pupil[2]	**£15**
**Classroom component only- Total Cost[3]**	**£72,650**
Mean cost/school[1]	**£692**
Mean cost/pupil[2]	**£13**
**Materials and teacher training only- Total Cost[4]**	**£44,773**
Mean cost/school[1]	**£426**
Mean cost/pupil[2]	**£8**

[1]based on 5749 pupils at baseline and 192 classes
[2]based on 105 schools
[3]excluding parental component cost of £13,250 for facilitator
[4] based on Stage 1 costs only

For peer review only

623 Table 3 Costs (£) at 33 months, by group.

	**Intervention** n=4189	**Education as normal** n=4037	**Difference (95% CI)**
Costs (£; mean; 95% CI)			
Education	287.46 (247.33, 327.58)	284.81 (244.20, 325.42)	2.65 (-54.29, 59.60)
Health	1839.07 (1564.87, 2113.26)	1906.55 (1627.59, 51)	-67.48 (-460.16, 325.19)
Criminal	128.16 (76.67, 179.66)	101.89 (49.43, 154.35)	26.27 (-47.55, 100.10)
Total Public Service Costs	2260.47 (1950.23, 2570.72)	2292.66 (1977.10, 2608.22)	-32.19 (-476.38, 412.00)

624 Costs discounted at 3.5%. Values are mean (95% confidence interval) adjusted for baseline covariates and
625 clustering. n, number analysed.

626

For peer review only

627 Table 4 Proportion of pupils reporting any heavy drinking episode in the previous 30 days at
628 33 months by group.

**Intervention** n=4189	**Education as normal** n=4037	Difference in effect (proportion of pupils reporting a heavy drinking episode in previous 30 days *avoided*) **(95% CI)***
0.15 (0.14, 0.17)	0.23 (0.21, 0.25)	0.08 (0.06, 0.09)

629 Values are proportion (95% confidence interval) adjusted for baseline covariates and clustering. n= number
630 analysed
631 * Confidence interval based on 1000 bootstrap resamples
632
633

For peer review only

634 Table 5 Results of the primary cost-effectiveness analysis and sensitivity analyses.

Analysis	Intervention, number analysed (% total)	Control, number analysed (% total)	Difference in total health service costs (£)	Difference in effect (proportion of pupils reporting a heavy drinking episode in previous 30 days *avoided*)
Primary analysis at 33 months	4189 (65.7)	4037 (63.5)	-17.19 (-402.84, 368.46)	0.08 (0.06, 0.09)
Sensitivity analysis for primary cost-effectiveness analysis at 33 months				
Multiple imputation for missing cost and outcome data	6379 (100)	6359 (100)	34.10 (-299.44, 367.44)	0.08 (0.07, 0.09)
Discounting costs and outcome at 1.5%	4189 (65.7)	4037 (63.5)	-17.63 (-410.62, 375.36)	0.07 (0.06, 0.09)
Adjustment of costs and outcome data for cluster only	4189 (65.7)	4037 (63.5)	-49.23 (-419.52, 321.07)	0.08 (0.06, 0.10)
5% increase in costs	4189 (65.7)	4037 (63.5)	-18.05 (-422.98, 386.88)	0.07 (0.06, 0.09)
5% decrease in costs	4189 (65.7)	4037 (63.5)	-16.33 (-382.70, 350.04)	0.07 (0.06, 0.09)

635 Total participants in the study= 12738 (Intervention=6379, Education as Normal=6359). I=intervention. EAN,
636 Education as Normal.
637

For peer review only

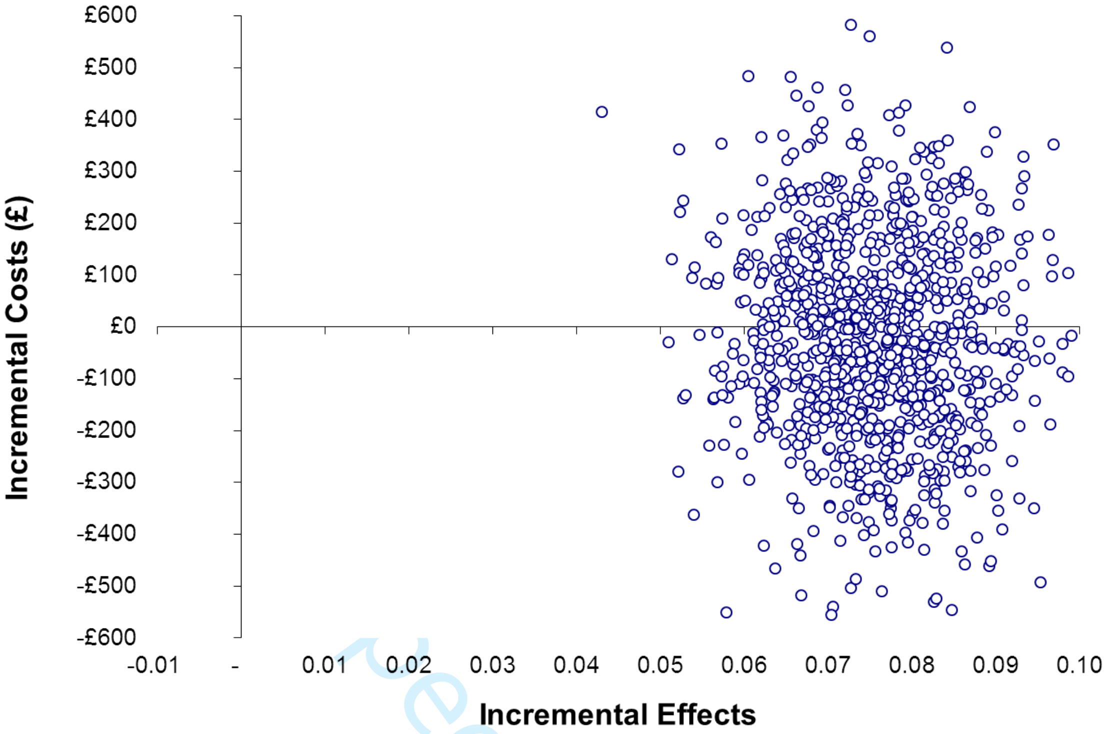

Figure 1 Cost-effectiveness plane for the primary cost-effectiveness analysis showing bootstrapped replications of mean incremental costs and pupils experiencing heavy drinking-avoided.

For peer review only

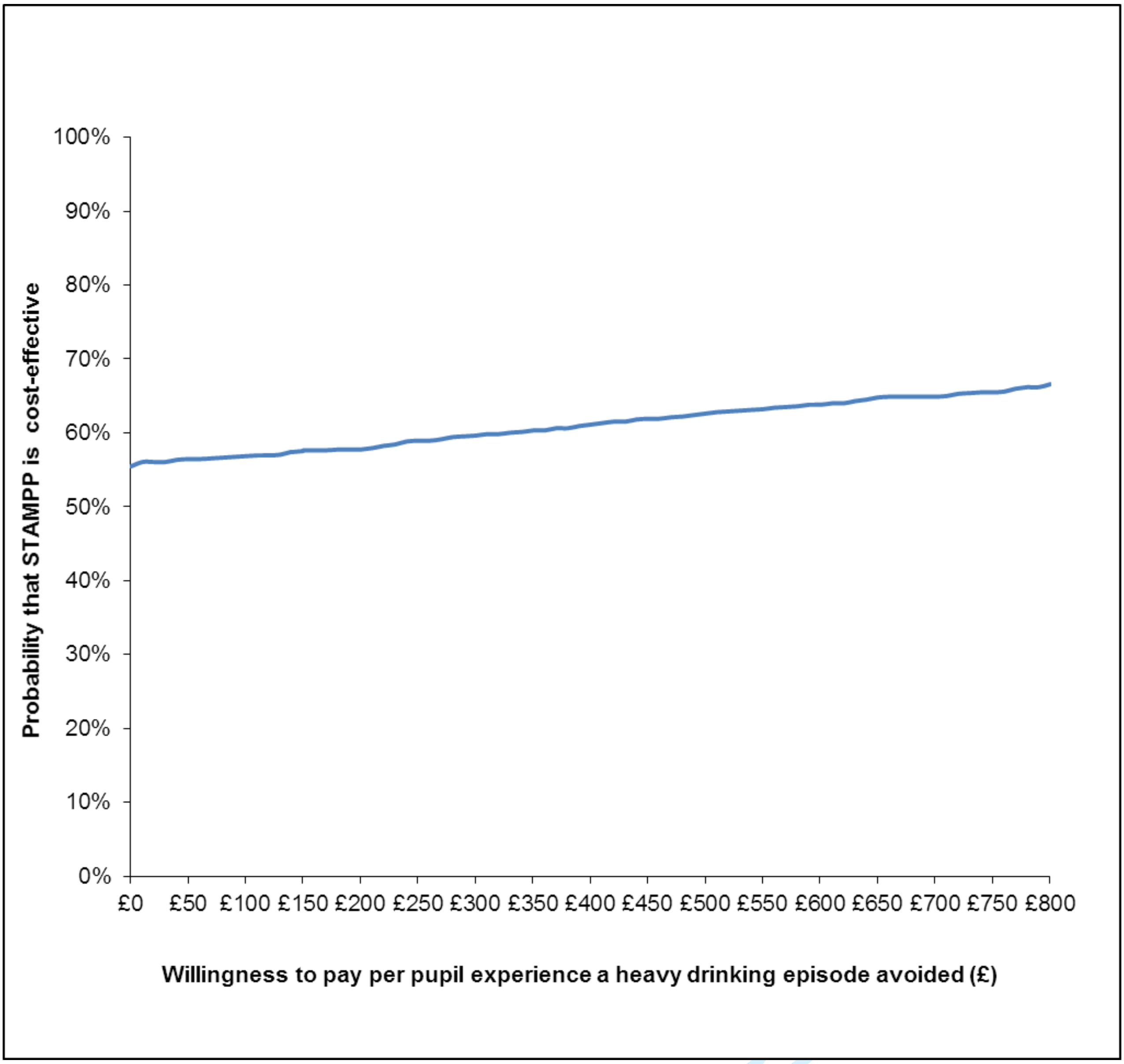

Figure 2 Cost effectiveness acceptability curve showing the probability of STAMPP being cost-effective compared to usual education.

Online Supplementary File

Table S1 Intervention resource use and costs

Resource use	Unit cost (£)	Number of units	Total cost (£)	Cost details
**Stage 1 Planning and preparation for delivery**				
**Intervention materials**				
Phase 1 Teacher Manual	n/a	145	240	16 pages
Phase 2 Teacher Manual	n/a	145	293	22 pages
Phase 1 Pupil Workbook	n/a	5749	2623	20 pages
Phase 2 Pupil Workbook	n/a	5749	2623	20 pages
Parent & Guardian Information Sheet	n/a	5749	255	single-sided page
Parent & Guardian Information Sheet (follow up)	n/a	5749	255	single-sided page
Phase 1 CD-ROM	1.40	145	203	cost of duplication
Phase 2 CD-ROM	1.40	145	203	cost of duplication
**Training of the teachers (Phase 1)**				
Northern Ireland				
Training	500.00	3	1500	Training delivered by a non-government organisation facilitator (£500 per day)
Teachers	161.79	107	17312	Based on 1 day (6.5 hours) teaching cover for a junior teacher at £24.89/hour
Location & associated cost (e.g. room hire)	25.00	107	2675	Based on local hotel costs including food & refreshments per head
Scotland				
Training	500.00	2	1000	Training delivered by a non-government organisation facilitator (£500 per day)
Teachers	161.79	38	6148	Based on 1 day (6.5 hours) teaching cover for a junior teacher at £24.89/hour
Location & associated cost (e.g. room hire)	25.00	38	950	Local hotel costs including food & refreshments (£25/head)

For peer review only

**Training of the teachers (Phase 2)**				
Northern Ireland				
Training sessions	500.00	3	1500	Training delivered by a non-government organisation facilitator (£500 per day)
Teachers	24.89	83	2066	based on 1 day (6.5 hours) teaching cover for a junior teacher
Location & associated cost (e.g. room hire)	28.78	83	2389	Local hotel costs including food & refreshments (£28.78/head)
Scotland				
Trainer	500.00	1	500	Training delivered by a non-government organisation facilitator (£500 per day)
Teachers	24.89	38	946	based on 1 day (6.5 hours) teaching cover for a junior teacher
Location & associated cost (e.g. room hire)	28.78	38	1094	Local hotel costs including food & refreshments (£28.78/head)
**Stage 2 Delivery**				
Phase 1 Teacher time input	14.52	6	87	Based on 35 minute lessons
Phase 2 Teacher time input	14.52	4	58	Based on 35 minute lessons
Parental component delivery	250.00	53	13250	Delivered by a non-government organisation facilitator (£500 per day)

For peer review only

Use of public sector services by all pupils with available data at each of the time points.

Table S2 Public sector service by treatment arm from 6 to 12 months. Values are number (percentages) of pupils using the service and mean (SD) use.

Service	Intervention (n=4189)		Control (n=4037)	
	Number using the service (%)	Mean (SD)	Number using the service (%)	Mean (SD)
**Education**				
School nurse	1792 (42.8)	0.9 (3.0)	1518 (37.6)	0.9 (3.4)
School counsellor/guidance teacher	642 (15.3)	0.5 (3.5)	646 (16.0)	0.4 (1.8)
Educational psychologist	71 (1.7)	0.0 (0.8)	84 (2.1)	0.0 (0.4)
Education welfare officer/ home-school liaison officer	59 (1.4)	0.0 (0.4)	89 (2.2)	0.0 (0.9)
**Health**				
GP surgery visit	2033 (48.5)	1.1 (5.0)	1791 (44.4)	0.9 (1.8)
GP out of hours	521 (12.4)	0.2 (0.9)	423 (10.5)	0.2 (0.8)
Nurse (other than school nurse)	839 (20.0)	0.3 (0.9)	705 (17.5)	0.3 (0.8)
Hospital appointment	1338 (31.9)	0.6 (1.5)	1200 (29.7)	0.6 (2.1)
Accident & Emergency	827 (19.7)	0.3 (0.7)	737 (18.3)	0.3 (1.1)
Overnight hospital stay	270 (6.5)	0.2 (2.0)	261 (6.5)	0.3 (3.7)
Psychologist	72 (1.7)	0.0 (0.8)	81 (2.0)	0.0 (0.5)
Counsellor (other than at school)	76 (1.8)	0.1 (0.9)	65 (1.6)	0.1 (1.4)
Social worker	142 (3.4)	0.1 (1.7)	112 (2.8)	0.1 (1.6)
Telephone help-line	30 (0.7)	0.0 (0.4)	46 (1.1)	0.0 (0.4)
**Criminal Justice**				
Youth justice service	32 (0.8)	0.0 (0.6)	33 (0.8)	0.0 (0.3)
Police service	108 (2.6)	0.0 (0.4)	120 (3.0)	0.0 (0.4)

For peer review only

Table S3 Public sector service by treatment arm from 18 to 24 months. Values are number (percentages) of pupils using the service and mean (SD) use.

Service	Intervention (n=4189)		Control (n=4037)	
	Number using the service (%)	Mean (SD)	Number using the service (%)	Mean (SD)
**Education**				
School nurse	1403 (33.5)	0.7 (2.2)	1325 (32.8)	0.7 (3.4)
School counsellor/guidance teacher	567 (13.5)	0.4 (3.0)	608 (15.1)	0.5 (3.3)
Educational psychologist	62 (1.5)	0.1 (1.9)	42 (1.0)	0.0 (0.5)
Education welfare officer/ home-school liaison officer	84 (2.0)	0.1 (1.3)	55 (1.4)	0.0 (0.5)
**Health**				
GP surgery visit	2013 (48.1)	0.9 (2.2)	1978 (49.0)	0.9 (1.4)
GP out of hours	498 (11.9)	0.2 (1.8)	462 (11.4)	0.2 (0.6)
Nurse (other than school nurse)	768 (18.3)	0.3 (2.2)	752 (18.6)	0.3 (1.0)
Hospital appointment	1269 (30.3)	0.6 (2.7)	1269 (31.4)	0.6 (0.8)
Accident & Emergency	764 (18.2)	0.3 (2.3)	810 (20.1)	0.3 (0.8)
Overnight hospital stay	245 (5.9)	0.3 (3.3)	266 (6.6)	0.3 (7.3)
Psychologist	86 (2.1)	0.1 (2.6)	91 (2.3)	0.1 (1.0)
Counsellor (other than at school)	111 (2.7)	0.1 (2.8)	104 (2.6)	0.1 (1.1)
Social worker	137 (3.3)	0.6 (3.3)	132 (3.3)	0.1 (1.2)
Telephone help-line	42 (1.0)	0.1 (3.0)	48 (1.2)	0.1 (0.9)
**Criminal Justice**				
Youth justice service	31 (0.7)	0.1 (3.2)	30 (0.7)	0.0 (1.0)
Police service	144 (3.4)	0.1 (3.3)	129 (3.2)	0.1 (1.6)

For peer review only

Table S4 Public sector service by treatment arm from 27 to 33 months. Values are number (percentages) of pupils using the service and mean (SD) use.

Service	Intervention (n=4189)		Control (n=4037)	
	**Number using the service (%)**	**Mean (SD)**	**Number using the service (%)**	**Mean (SD)**
**Education**				
School nurse	1195 (28.5)	0.6 (2.2)	1094 (27.1)	0.5 (1.4)
School counsellor/guidance teacher	558 (13.3)	0.6 (3.7)	560 (13.9)	0.6 (3.8)
Educational psychologist	41 (0.98)	0.0 (0.4)	39 (1.0)	0.0 (0.4)
Education welfare officer/ home-school liaison officer	64 (1.5)	0.1 (4.5)	56 (1.4)	0.0 (0.5)
**Health Sector**				
GP surgery visit	1943 (46.4)	1.0 (1.8)	1790 (44.3)	1.0 (2.2)
GP out of hours	399 (9.5)	0.1 (0.6)	350 (8.7)	0.1 (0.7)
Nurse (other than school nurse)	622 (14.9)	0.3 (0.9)	593 (14.7)	0.3 (1.0)
Hospital appointment	1073 (25.6)	0.6 (1.7)	975 (24.2)	0.5 (2.0)
Accident & Emergency	635 (15.2)	0.2 (0.6)	626 (15.5)	0.2 (1.0)
Overnight hospital stay	169 (4.0)	0.1 (1.0)	161 (4.0)	0.1 (1.0)
Psychologist	85 (2.0)	0.1 (1.5)	73 (1.8)	0.1 (0.8)
Counsellor (other than at school)	96 (2.29)	0.2 (1.6)	98 (2.4)	0.1 (1.6)
Social worker	85 (2.03)	0.1 (1.3)	108 (2.7)	0.1 (1.1)
Telephone help-line	43 (1.0)	0.0 (0.6)	39 (1.0)	0.0 (0.4)
**Criminal Justice Service**				
Youth justice service	10 (0.2)	0.0 (0.3)	18 (0.5)	0.0 (0.3)
Police service	97 (2.3)	0.1 (0.5)	111 (2.8)	0.0 (0.6)

For peer review only

Figure S1

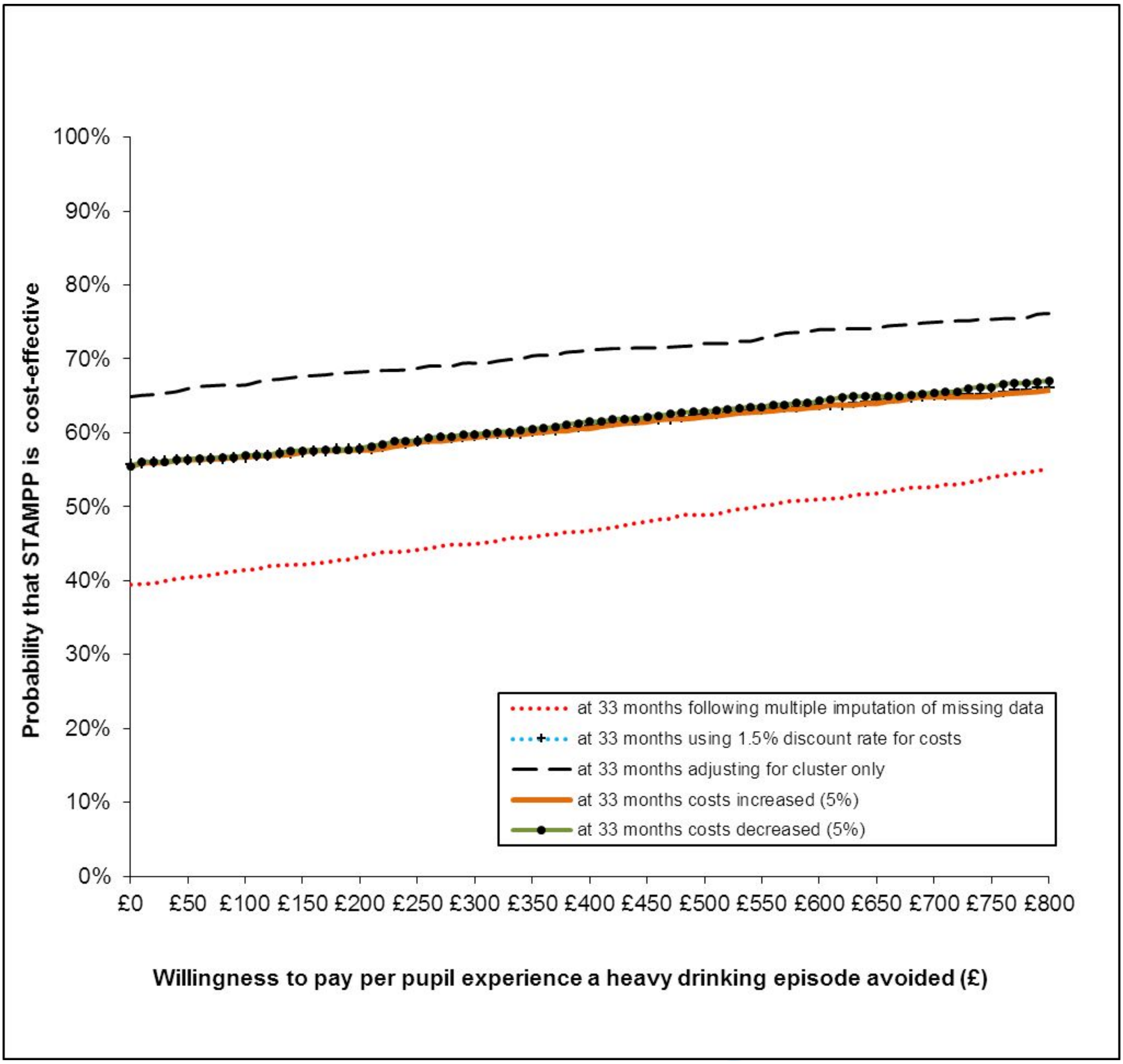

Figure S1 Cost effectiveness acceptability curves showing the probability of STAMPP being cost-effective compared to usual education for the sensitivity analyses.

Consolidated Health Economic Evaluation Reporting Standards (CHEERS) Checklist

Items to include when reporting economic evaluations of health interventions

Section/Topic	Item No	Recommendation	Reported on page No / Line No
**Title and abstract**			
Title	1	Identify the study as an economic evaluation or use more specific terms such as "cost-effectiveness analysis", and describe the interventions compared.	**Line 1**
Abstract	2	Provide a structured summary of objectives, perspective, setting, methods (including study design and inputs), results (including base case and uncertainty analyses), and conclusions.	**Lines 6-36**
**Introduction**			
Background and objectives	3	Provide an explicit statement of the broader context for the study.	**Lines 63-66**
		Present the study question and its relevance for health policy or practice decisions.	**Lines 90-91**
**Methods**			
Target population and subgroups	4	Describe characteristics of the base case population and subgroups analysed, including why they were chosen.	**Lines 94-97**
Setting and location	5	State relevant aspects of the system(s) in which the decision(s) need(s) to be made.	**Lines 95 -96**
Study perspective	6	Describe the perspective of the study and relate this to the costs being evaluated.	**Lines 99-101**
Comparators	7	Describe the interventions or strategies being compared and state why they were chosen.	**Lines 94- details of intervention in main trial paper**
Time horizon	8	State the time horizon(s) over which costs and consequences are being evaluated and say why appropriate.	**Line 99**
Discount rate	9	Report the choice of discount rate(s) used for costs and outcomes and say why appropriate.	**Lines 218-219, lines 233-234**
Choice of health outcomes	10	Describe what outcomes were used as the measure(s) of benefit in the evaluation and their relevance for the type of analysis performed.	**Lines 104-108**
Measurement of effectiveness	11a	*Single study-based estimates:* Describe fully the design features of the single effectiveness study and why the single study was a sufficient source of clinical effectiveness data.	**Full details of trial in main paper**
	11b	*Synthesis-based estimates:* Describe fully the methods used for identification of included studies and synthesis of clinical effectiveness data.	**n/a**
Measurement and valuation of preference-	12	If applicable, describe the population and methods used to elicit preferences for outcomes.	**n/a**

For peer review only

based outcomes			
Estimating resources and costs	13a	*Single study-based economic evaluation:* Describe approaches used to estimate resource use associated with the alternative interventions. Describe primary or secondary research methods for valuing each resource item in terms of its unit cost. Describe any adjustments made to approximate to opportunity costs.	**Line 112-188**
	13b	*Model-based economic evaluation:* Describe approaches and data sources used to estimate resource use associated with model health states. Describe primary or secondary research methods for valuing each resource item in terms of its unit cost. Describe any adjustments made to approximate to opportunity costs.	**n/a**
Currency, price data, and conversion	14	Report the dates of the estimated resource quantities and unit costs. Describe methods for adjusting estimated unit costs to the year of reported costs if necessary. Describe methods for converting costs into a common currency base and the exchange rate.	**Lines 177-178, Table 1**
Choice of model	15	Describe and give reasons for the specific type of decision-analytical model used. Providing a figure to show model structure is strongly recommended.	**n/a**
Assumptions	16	Describe all structural or other assumptions underpinning the decision-analytical model.	**n/a**
Analytic methods	17	Describe all analytical methods supporting the evaluation. This could include methods for dealing with skewed, missing, or censored data; extrapolation methods; methods for pooling data; approaches to validate or make adjustments (such as half cycle corrections) to a model; and methods for handling population heterogeneity and uncertainty.	**Lines 191-240**
**Results**			
Study parameters	18	Report the values, ranges, references, and, if used, probability distributions for all parameters. Report reasons or sources for distributions used to represent uncertainty where appropriate. Providing a table to show the input values is strongly recommended.	**n/a**
Incremental costs and outcomes	19	For each intervention, report mean values for the main categories of estimated costs and outcomes of interest, as well as mean differences between the comparator groups. If applicable, report incremental cost-effectiveness ratios.	**Table 2, Table 3, Table 4, Table 5**
Characterizing uncertainty	20a	*Single study-based economic evaluation:* Describe the effects of sampling uncertainty for the estimated incremental cost and incremental effectiveness parameters, together with the impact of methodological assumptions (such as discount rate, study perspective).	**Table 5 line 289-307**
	20b	*Model-based economic evaluation:* Describe the effects on the results of uncertainty for all input parameters, and uncertainty related to the structure of the model and assumptions.	**n/a**
Characterizing heterogeneity	21	If applicable, report differences in costs, outcomes, or cost-effectiveness that can be explained by variations between subgroups of patients with different baseline characteristics or other observed variability in effects that are not reducible by more information.	**n/a**
**Discussion**			
Study findings, limitations, generalizability, and current knowledge	22	Summarise key study findings and describe how they support the conclusions reached. Discuss limitations and the generalisability of the findings and how the findings fit with current knowledge.	**Lines 311-402**
**Other**			
Source of funding	23	Describe how the study was funded and the role of the funder in the identification, design, conduct, and reporting of the analysis. Describe other non-monetary sources of support.	**Lines 421-426**
Conflicts of interest	24	Describe any potential for conflict of interest of study contributors in accordance with journal policy. In the absence of a journal policy, we recommend authors comply with International Committee of Medical Journal Editors recommendations.	**Lines 458-465**

For peer review only

For peer review only

BMJ Open

Cost-effectiveness of a combined classroom curriculum and parental intervention: economic evaluation of data from the Steps Towards Alcohol Misuse Prevention Programme (STAMPP) cluster randomised controlled trial.

Journal:	*BMJ Open*
Manuscript ID	bmjopen-2018-027951.R1
Article Type:	Research
Date Submitted by the Author:	30-Apr-2019
Complete List of Authors:	Agus, Ashley; Northern Ireland Clinical Trials Unit, Health Economics<br>McKay, Michael; University of Liverpool School of Life Sciences, Psychological Sciences<br>Cole, Jonathan; University of Liverpool<br>Doherty, Paul; Northern Ireland Clinical Trials Unit<br>Foxcroft, David; Oxford Brookes University UK<br>Harvey, Séamus<br>Murphy, Lynn; Northern Ireland Clinical Trials Unit<br>Percy, Andrew; Queen's University Belfast, Social Sciences, Education and Social Work<br>Sumnall, Harry; Liverpool John Moores University, Public Health Institute
**Primary Subject Heading**:	Health economics
Secondary Subject Heading:	Public health
Keywords:	PUBLIC HEALTH, HEALTH ECONOMICS, EDUCATION & TRAINING (see Medical Education & Training), Community child health < PAEDIATRICS, Alcohol misuse, School-based

SCHOLARONE™
Manuscripts

Cost-effectiveness of a combined classroom curriculum and parental intervention: economic evaluation of data from the Steps Towards Alcohol Misuse Prevention Programme (STAMPP) cluster randomised controlled trial.

Corresponding author: Ashley Agus[1]

Email: ashley.agus@nictu.hscni.net

Authors:

Ashley Agus[1], Michael McKay[2], Jonathan Cole[2], Paul Doherty[1], David R. Foxcroft[3], Séamus Harvey[4], Lynn Murphy[1], Andrew Percy[5], Harry Sumnall[6]

[1] Northern Ireland Clinical Trials Unit, The Royal Hospitals, Belfast, UK

[2] Department of Psychological Sciences, University of Liverpool, UK

[3] Psychology and Public Health, Oxford Brookes University, UK

[4] School of Sport, Health and Exercise Sciences, University of Bangor, UK

[5] School of Social Sciences, Education, and Social Work, Queen's University Belfast, UK

[6] Public Health Institute, Liverpool John Moores University, UK

Abstract (word count 294)

Objectives: To assess the cost-effectiveness of the Steps Towards Alcohol Misuse Prevention Programme (STAMPP) compared with education as normal (EAN) in reducing self-reported heavy episodic drinking in adolescents.

For peer review only

Design: A cost-effectiveness analysis from a public sector perspective conducted as part of a cluster randomised trial

Setting: 105 High schools in Northern Ireland (NI) and in Scotland.

Participants: Students in school year 8/S1 (aged 11-12) at baseline.

Interventions: A classroom-based alcohol education curricula, combined with a brief alcohol intervention for parents/carers.

Outcome measures: Cost per young person experiencing heavy episodic drinking avoided due to STAMPP at 33 months from baseline.

Results: The total cost of STAMPP was £85,900, equivalent to £818 per school and £15 per pupil. Due to very low uptake of the parental component, we calculated costs of £692 per school and £13 per pupil without this element. Costs per pupil were reduced further to £426 per school and £8 per pupil when it was assumed there were no additional costs of classroom delivery if STAMPP was delivered as part of activities such as Personal, social, health and economic education (PSHE). STAMPP was associated with a significantly greater proportion of pupils experiencing a heavy drinking episode avoided (0.08/8%) and slightly lower public sector costs (mean difference -£17.19). At a notional willingness-to-pay threshold of £15 (reflecting the cost of STAMPP) the probability of STAMPP being cost-effective was 56%. This level of uncertainty reflected the substantial variability in the cost differences between groups.

Conclusions: STAMPP was relatively low-cost and reduced heavy episodic drinking. STAMPP was not associated with any clear public sector cost-savings, but neither did it increase them or lead to any cost-shifting within the public sector categories. Further research is required to establish if the cost-effectiveness of STAMPP is sustained in the long-term.

Trial registration: ISRCTN47028486. The date of registration was 23/09/2011, and school recruitment began 01/11/2011.

Strengths and limitations of this study

- A broad public sector perspective was adopted for the analysis.
- Participant level service use data was obtained directly from the students thus avoiding the need to involve parents/ guardians in questionnaire completion.
- Study was not specifically powered to detect statistically significant differences in costs or cost-effectiveness.

For peer review only

Only two-thirds of the students had complete cost and outcome data

Introduction

Although the prevalence of alcohol use amongst children and young people is generally decreasing in the United Kingdom (UK), it is high in comparison to many European countries.[1-5] Children and young people who engage in heavy drinking expose themselves to a wide range of negative physical and mental health, social, legal and educational risks.[6-9] As alcohol consumption patterns established during adolescence appear to persist well into adulthood,[10,11] heavy drinking in adolescence may contribute to increased health and social services utilisation, placing a substantial economic burden on society.[12,13] Indeed, considering the external costs imposed on society only, the annual burden of alcohol for England and Wales, Scotland, and Northern Ireland (NI) are reportedly £21 billion, £2.1 billion and £680 million respectively.[14] Heavy episodic (binge) drinking costs alone have been estimated at £4.86 billion per year (£77 per capita at 2014 prices).[12] Given the complexity and scale of the problem, multiple system-wide interventions are required to address the burden of alcohol-related harm, including cost-effective prevention interventions to reduce binge drinking in young people.

In addition to environmental prevention and policy led approaches to alcohol (e.g. pricing and marketing restrictions),[15] systematic reviews suggest that some universal alcohol prevention interventions are effective in reducing use in young people.[16] Although existing National Institute for Health and Care Excellence (NICE) guidelines on school-based alcohol prevention (update expected 2019) do not recommend any single prevention programme, they called for partnership working between schools and other stakeholders in efforts to prevent use.[17] NICE suggested that school-based interventions should aim to increase knowledge about alcohol, to explore perceptions about alcohol use, and to help develop decision-making skills, self-efficacy and self-esteem. The guideline also highlighted the paucity of evidence from economic evaluations of school-based programmes, although a few additional studies have subsequently emerged (see Hill et al., 2017[18] for a recent review).

The Steps Towards Alcohol Misuse Prevention Programme (STAMPP) cluster randomised controlled trial was undertaken to assess the effectiveness of a combined school-based universal alcohol harm reduction curriculum and a brief parental intervention, compared with education as normal (EAN)[19,20,21] in reducing self-reported heavy episodic drinking (HED) and alcohol-related harms (ARHs) in adolescents. Heavy episodic drinking was defined as the consumption of $\geq$ 6 units in a single episode for male students and $\geq$ 4.5 units for female

For peer review only

students. Examples of ARHs include getting into fights, damaging property or poorer school performance. The trial found that the STAMPP intervention was well received by both pupils and school staff and it reduced self-reported HED in the past 30 days at 33 month follow-up with statistically significantly fewer students reporting HED in the intervention group compared with EAN. The intervention did not reduce ARHs associated with own drinking, which were low in both trial arms. There was low uptake of the parental component which comprised a presentation delivered by a trained facilitator at school-based parent evenings, followed up by an information leaflet and survey mailed to all intervention pupils' parents. Only 9% of eligible parents in NI and 2.5% of eligible parents in Scotland attended the evening and only 31% in NI and 18% in Scotland returned the survey. Despite this low uptake, it was uncertain whether the observed intervention effect on HED could be accounted for entirely by the classroom component.

The aim of this paper is to report on the cost-effectiveness of STAMPP compared with EAN in reducing self-reported heavy episodic drinking in adolescents.

Methods

The STAMPP trial has been described in detail elsewhere.[19,20] It recruited 12,738 participants (Intervention=6379, Education as Normal=6359) from 105 schools in NI and the Glasgow and Inverclyde areas of Scotland. All participants were in year 8/S1 (aged 11-12) at baseline (June 2012). We performed an incremental cost-effectiveness analysis alongside the trial to estimate the cost per young person experiencing heavy episodic drinking avoided due to STAMPP at 33 months. A public sector perspective was adopted for the analysis, which encompassed the costs to Local Authorities, National Health Service (NHS), Personal Social Services (PSS) and Criminal Justice Service.

Outcomes

Consistent with the primary outcome of the study, the primary effectiveness measure was the number of pupils who reported any HED in the previous 30 days at 33 months. This was based on responses to the question *"How often in the past month have you drank 4.5 (female) / 6 (male) or more units of alcohol?"* Descriptive statistics were used to summarise the proportion of pupils in each arm reporting a heavy drinking episode.

Costs

For peer review only

Intervention resources use

We calculated the economic cost of STAMPP according to the principle of opportunity cost, that is, we attempted to place a value on the benefits which were foregone by STAMPP being delivered instead of something else. We therefore included the full value of all the resources it used, regardless of whether the resources were directly purchased for the study. The process evaluation carried out alongside the trial established that STAMPP was delivered in most schools as part of their personal, social, health and economic (PSHE) (or local equivalent) provision and that curriculum-based alcohol education activities were found to be minimal in both EAN and intervention schools.[19] We therefore made the very conservative assumption that there were no costs associated with EAN, that is, STAMPP was seen as an additional cost to EAN and the costs of other alcohol education activities were not included in the analysis.

Resources were categorised according to the stage they were used in the research process; planning and preparation for delivery (stage 1), and delivery itself (stage 2), in keeping with other trials of behavioural intervention.[22-24] Pre-start-up resources associated with the development of STAMPP were not included in the analyses as they would not be incurred should the intervention be incorporated in to the curriculum in the future. These included the development of the teacher manual and pupil work book content, planning the lessons and the design of materials. Further details are provided in the online supplementary file.

Students' service use

Data on service use by all pupils from baseline to 33 months was collected using an instrument administered at baseline, 12 months, 24 months and 33 months.[19] The instrument incorporated some items taken from the Client Service Receipt Inventory[25] specifically adapted for childhood[26] and items relating to the use of judicial services. An information page was provided containing definitions of some of the public services in case the students were unfamiliar with them. The instrument was designed with input from relevant professionals (e.g. educational psychologist, social workers, Scottish and NI teachers) and reviewed by a social researcher experienced in delivering questionnaires to children, and other health economists. The instrument asked pupils to report their use of services in the previous six months thus providing service use data for; the 6 months pre-baseline, 7-12 months, 19-24 months and 28-33 months. If there were any missing fields within the service use questionnaire, it was assumed that the relevant service had not been used.

For peer review only

Individual-level service use was combined with unit costs (Table 1) to estimate a cost for each pupil for each of the four survey time periods. Unit costs were obtained from publicly available sources[27-29] and set at 2013-14. For school counsellors/guidance teacher we assumed a mid-point of on the Upper Pay Scale (UPS2)[30] and included 24% for employers' costs. As there were gaps in the survey period where no cost data were collected (baseline to 6 months, 12-18 months and 24-27 months), we used interpolation of the available cost data to fill in these gaps. This was done separately for each participant to obtain an estimate of their total service use costs at 33 months. Total costs could not be calculated for those pupils who had any missing cost data points as these were required for the interpolation. Descriptive statistics were used to summarise service use and costs at each time point for the different categories of the public sector (Education, Health and Criminal Justice). Costs of service use in the 6 months before baseline were not included in total costs but were used in the interpolation.

[Table 1]

Cost-effectiveness analysis

Cost-effectiveness was estimated using conventional decision rules[31] and reported as incremental cost-effectiveness ratios (ICER) if appropriate. The ICER is a measure of the additional cost per additional unit of effect produced by one intervention compared with another. For an intervention to be considered cost-effective, the ICER must be less than the maximum amount of money that a decision-maker would be willing to pay per unit of effect, that is, their willingness-to-pay (WTP) threshold. A cost-effectiveness analysis (CEA) estimated the incremental cost per young person experiencing HED avoided due to STAMPP at 33 months. To maintain the correlation structure of the data, only pupils with complete cost and outcome data were included in the analyses. This meant pupils with cost data from baseline to 33 months and a response to the primary outcome variable at 33 months.

Bootstrapped multilevel mixed-effects regression models were used to estimate mean incremental costs and effects (with 95% confidence intervals based on 1000 bootstrap resamples). Both models adjusted for school location (NI/ Scotland), school level of FSM provision (Low; 0-15.4%, Moderate; 15.5-30.4%, High; 30.5% and above), school type (all boys' school/ all girls' school/ co-education school) and clustering. In addition, the cost model adjusted for pupil's baseline costs and the effects model adjusted for pupils' baseline drinking.

For peer review only

Uncertainty in the cost-effectiveness measures was investigated by using the 1000 resampled incremental costs and effects to generate 1000 replications of the ICERs. The replicates were plotted on the cost-effectiveness plane and used to construct cost-effectiveness acceptability curves (CEACs). The CEACs showed the probability of STAMPP being cost-effective compared with EAN at different threshold levels of WTP to avoid a pupil experiencing an episode of heavy drinking in the previous 30 days at 33 months. As there is no generally accepted threshold value for cost per pupil experiencing any HED avoided we looked at range of thresholds, including the cost of the intervention per pupil, and compared our findings with those of other economic evaluations which have been performed in this research area.

All analyses were performed using Stata 12/IC for Windows®. Costs occurring in the second and third year of the study were discounted at 3.5% in keeping with NICE (2013) guidance.[32]

Sensitivity analyses

A number of one-way sensitivity analyses were performed to test the robustness of the cost-effectiveness findings;

- Missing total costs and outcomes were filled simultaneously using multiple imputation by chained equations. In the multiple imputation model we included all of the variables which were to be included in the subsequent multilevel models i.e. the treatment variable, baseline costs, baseline drinking, school location, school level of entitlement to free meals and clustering. We also included 6-12 month costs and 6-12 month drinking. We used predictive mean matching for costs and a logit model for the primary outcome. Five imputed data sets were generated and the results combined.

- Total costs were discounted at a rate of 1.5% as suggested by NICE[33] for public health interventions.

- The multilevel models were re-estimated without adjusting for baseline covariates but still adjusting for clustering.

- Since a linear time trend was assumed between data time points this might have led to total costs and total heavy drinking episodes being under/over-estimated if said trend is not appropriate. The impact of increasing and decreasing total costs by 5% were therefore explored.

For peer review only

Results

A total number of 12,738 pupils took part in the study, with 6379 in the intervention group and 6359 in usual education. There were 11,316 pupils present at the baseline assessment; 5749 in the intervention group and 5567 in EAN. Approximately two-thirds of all pupils had complete cost and outcome data and were included in the analysis. This was similar across groups (Intervention =66%; 4189/6379) (EAN=63%; 4037/6359). Of those pupils not included in the analysis (n=4512), 97% had missing cost data, 56% had missing outcome data and 53% were missing both (see Table S1, online supplementary file).

The resources and costs used in the planning and preparation, and delivery of the intervention are presented in Table S2 of the online supplementary file.. Total costs are presented in Table 2. The mean cost per school was £818 and the mean cost per pupil was £15. The largest proportion of the costs was associated with the training of the teachers as this involved teaching cover, location costs and facilitator costs. The second largest cost was associated with the delivery of the intervention in the classroom setting.

[Table 2]

The use of public sector services by all pupils with available data in the 6 months pre-baseline, from 6 to 12 months, from 18-24 months and from 27 to 33 months are presented in the online supplementary file (Tables S3-S5). For those pupils with complete data, the costs of public services used over the study period following interpolation of costs and adjustment for baseline covariates and clustering are shown in Table 3. Negative costs reflect a cost saving in favour of the intervention. The difference in total public sector costs was small (£ and not statistically significant.

[Table 3]

The proportion of pupils reporting a heavy drinking episode in the previous 30 days is shown in Table 4. The outcomes are reported in terms of cases avoided; thus, a positive difference reflects a smaller number of pupils in the intervention arm experiencing a heavy drinking episode in the previous 30 days. Statistically significantly fewer pupils in the intervention arm reported drinking heavily in the previous 30 days (15 % vs. 23%) compared to EAN.

For peer review only

[Table 4]

Cost-effectiveness analysis

The results from the primary CEA are shown in Table 5. The cost per pupil for the intervention (£15) was added to each pupil in the intervention arm. STAMPP was associated with a statistically significantly greater proportion of pupils experiencing a heavy drinking episode *avoided* (0.08/8%; 0.06, 0.09) and lower mean total costs (-£17.19; -402.84, 368.46). The difference in costs was small and not statistically significant, therefore STAMPP could be considered cost-neutral. When an ICER is negative, as in this case (-17.19/0.08), its magnitude does not convey any meaning so they are not calculated.[34] STAMPP can be said to dominate usual education; however, since the difference in costs was not statistically different, only weak dominance can be claimed.[35] Uncertainty surrounding the estimates of total costs and outcomes is represented by the bootstrapped ICERs on the cost-effectiveness plane (

Figure 1). The majority of points straddle both the north east and south east quadrant indicating that, although STAMPP is likely to be more effective than the usual education, there is considerable variability about the cost estimates. The corresponding CEAC is in Figure 2; when WTP thresholds ranging from £0 to £800 are considered, it can be seen that the probability of STAMPP being cost-effective compared to usual education ranges from 55% to 67%. Uncertainty in the cost-effectiveness of the intervention remains substantial until much higher WTP values, with an 80% probability being displayed at a WTP of £2,000. If decision-makers were only willing to pay the £15 (the cost of STAMPP per pupil) the probability would be 56%.

The results of the sensitivity analyses for the primary CEA are presented in Table 5 and the corresponding CEACs are in the online supplementary file (Figure S1). After the multiple imputation of missing data, the probability of STAMPP being cost-effective was lower at each WTP threshold, ranging from 40-55%. In light of this, we explored the baseline characteristics of pupils with complete and incomplete data (Table S6-S7 online supplementary file). Those with missing data were more likely to go to a school with a high proportion of free school meals, more likely to have reported HED in the previous 30 days at baseline and reported marginally higher public service use in the six months pre-baseline, compared with those pupils with complete data.

For peer review only

When the multilevel models were re-estimated adjusting only for baseline covariates the probability of cost-effectiveness was consistently higher, ranging from 65%-76%. Reducing the discount rate to 1.5% and increasing/decreasing costs by 5% had little effect on the cost-effectiveness of STAMPP.

[Table 5]

Discussion

The total cost to deliver STAMPP was £85,900, equivalent to £818 per school and £15 per pupil. In a review of economic evidence for the development of the NICE guidance[17] the cost of other effective school-based interventions range from £20- £150 (cost year 2005/6)[36-38] one of which was Australian School Health and Alcohol Harm Reduction Project (SHAHRP) from which the classroom component of STAMPP was adapted.[36] Another review of school-based skills development substance use prevention curricula in the USA (which targeted both alcohol and illicit drug estimated programme unit costs between US $100-400 per pupil[39]). Thus, at a cost of £15 per pupil, STAMPP is a relatively low-cost intervention that successfully reduces problematic drinking. If costs were extrapolated to the 685,300 students aged 12 years old in the UK in 2013/14 the cost would be £10.3 million in total. This is a relatively small amount compared with the overall annual economic burden from alcohol consumption in the UK. Furthermore, the cost per pupil could reduce to £8 per pupil if it was entirely classroom based and there was no additional cost for teachers' time to deliver it (see online supplement Table S8). This is justifiable considering the low uptake of the parental component and since we made the conservative assumption that STAMPP was delivered in addition to EAN; in reality, STAMPP is likely to have replaced whatever statutory provision was in place, however minimal, and potentially saved teachers' time as observed in a recent cRCT of a school-based sexual education intervention.[24]

Costs were estimated from a public sector perspective, which was justified considering one of the principal objectives of STAMPP was to reduce ARHs in teenagers. It was hypothesised that this would in turn reduce the use of health and judicial services and the need for additional support within the school setting to address behavioural and emotional problems. The analysis of public service costs however showed only a small difference between groups at 33 months. This is in keeping with the analysis of the ARH data from the trial as no difference were observed in the number of self-reported ARHs by pupils between groups and indeed both groups reported low levels of harms overall. [19, 20]

For peer review only

Compared to EAN, significantly fewer pupils in the intervention group reported experiencing a heavy drinking episode in the previous 30 days at 33 months. The CEA indicated that STAMPP weakly dominated EAN. At a notional WTP threshold of £15 (reflecting the cost of STAMPP) the probability of STAMPP being cost-effective was 56%. This level of uncertainty reflects the considerable variability in the cost differences between groups.

The sensitivity analyses indicated that the results of the CEA were robust to small changes in parameters i.e. discounting and small increases in cost and effectiveness. However, when costs and effects were not adjusted for baseline covariates, the probability of cost-effectiveness of STAMPP increased. This suggests that the cost-effectiveness of STAMPP may vary between sub-groups and warrants further investigation to identify which pupils or schools might benefit the most from receiving the intervention. Furthermore, when multiple imputation was used to impute missing cost and outcome data the cost-effectiveness of STAMPP decreased. Multiple imputation is based on the assumption that data is missing at random, however a post hoc comparison of the baseline characteristics of pupils with complete and incomplete data indicated the assumption might not have been met. Pupils with incomplete data were more likely to attend a school with a high proportion of free school meals, more likely to report HED at baseline and reported higher public service use at baseline, compared to those with complete data.

The strengths of the economic evaluation include the choice of perspective; we adopted a public sector perspective in keeping with NICE guidance for public health interventions[33] thus we looked beyond the healthcare system and considered the broader impact of the intervention. In contrast, Hill et al (2017)[18] found that the majority of economic evaluations of alcohol prevention considered only health care costs; the danger being that interventions may be undervalued if cost-savings occur in other sectors or overvalued if costs are incurred in other sectors. A further strength was that we obtained resource use data directly from the pupils thus avoiding the need to involve parents/guardians in questionnaire completion. We provided definitions of the services using age appropriate terminology, with input from relevant professionals. Considering the difficulty associated with engaging parents/guardians in the study, reflected in the poor attendance to the parental evenings,[19] it is likely that a reliance on parents/guardians would have led to considerable amounts of missing data.

For peer review only

The study had a number of limitations. We relied on self-report to collect our outcomes, which may have led to under or over reporting of alcohol use through memory, social desirability and other biases.[40] However, adolescent self-reported alcohol questionnaires are generally reliable[41] and a low level (9.9%) of recanting (the denial of a previous positive report of lifetime[42]) was observed in this study.[43] Furthermore, all pupils completed the same questionnaires, so if bias had existed this would arguably have been equivalent between trial arms. In terms of self-report service use, cost data was more likely to be missing than outcome data. This likely reflects the need for pupils to complete multiple questionnaires over the study period in order to calculate total costs, whereas only a response to a single question at the final time point was required for outcome data.

The study was not specifically powered to detect statistically significant differences in costs or cost-effectiveness. Although CEA does not typically make decisions based on significance rules,[44] having a sufficiently powered study will allow decision-makers to be more confident in the value claim.[34] The resources used during the planning, preparation and delivery of the intervention were largely recorded retrospectively and costs were obtained from invoices when these were available. We endeavoured to use plausible assumptions when actual data were not available, but the consistent and prospective collection of resource use and costs would be lead to more robust data. We included pupils in the CEA only if they had complete cost and effect data. As a result, only two-thirds of the pupils were included in the analysis, the rest we assumed were missing at random. As discussed earlier, this assumption may be flawed. We performed a within trial cost-effectiveness analysis which is limited to the time horizon of the study (33months). In light of the literature linking excess and early initiation of drinking in adolescence to alcohol use behaviours in adulthood (e.g., the development of poor health outcomes over a sustained period of time,[45] it is important to investigate if the (cost) effectiveness of STAMPP is maintained or even increases in the long-term. A decision model was beyond the scope of this current study; however, it would provide a framework to explore the long-term impact of STAMPP. Finally, we did not undertake a formal analysis of the impact of STAMPP on health inequalities, but an equity impact analysis[46] that disaggregates costs and outcomes by equity-relevant sub groups such as gender, receipt of free school meals, school area deprivation, and ethnicity may help us to understand this impact better.

Conclusions

For peer review only

STAMPP was a relatively low-cost intervention that successfully reduced HED. STAMPP did not bring about clear public sector cost-savings; however, neither did it increase them or lead to any cost-shifting within the public sector categories. STAMPP can therefore be considered to weakly dominate EAN because it was both cost-neutral and more effective. Further research is required to establish if the cost-effectiveness of STAMPP is sustained in the long-term.

Acknowledgements

As well as acknowledging the role played by participating schools and school children, the authors would like to acknowledge the support of the following people in this project: Séamus Mullin, Gerry Bleakney, Owen O'Neill (PHANI); Malachy Crudden (CCMS), Maura Kearney, and Fergal Doherty (Psychological Services, Glasgow); Kate Watson (Psychological Services, Inverclyde) and John Butcher and Sandy Cunningham (Education Services, Glasgow). Thank you also to Rita Faria (Centre for Health Economics, University of York) for her valuable feedback on an early draft of the manuscript. The views and opinions expressed therein are those of the authors and do not necessarily reflect those of the NIHR-PHR, NIHR, NHS or the Department of Health.

Contributions: Sumnall had full access to all of the data in the study and takes responsibility for the integrity of the data and the accuracy of the data analysis. Agus undertook the health economic analysis, wrote the first draft of the manuscript and subsequent versions, and submitted the final version; Sumnall was project PI, contributed to the first draft and subsequent iterations of the manuscript; Cole, Doherty, Foxcroft Harvey, McKay, Murphy, Percy all contributed to drafts and approved the submission.

Funding: This trial was funded by the National Institute of Health Research (NIHR) Public Health Research (PHR) programme (project number 10/3002/09). The Public Health Agency of NI and Education Boards of Glasgow/Inverclyde provided some intervention costs. Diageo provided funds to print classroom workbooks for use only in the Glasgow Local Authority area. Remaining intervention costs were internally funded.

Disclaimer: The views and opinions expressed therein are those of the authors and do not necessarily reflect those of the NIHR-PHR, NIHR, NHS or the Department of Health. The research and intervention funders had no involvement in intervention design; design and

For peer review only

conduct of the study; collection, management, analysis, and interpretation of the data; and preparation, review or approval of the manuscript.

List of abbreviations

ARH- Alcohol Related Harms

CEA -Cost-effectiveness analysis

CEAC - Cost-effectiveness acceptability curves

EAN- Education as normal

HED- Heavy episodic drinking

ICER- Incremental cost-effectiveness ratios

NICE- National Institute for Health and Care Excellence

NHS- National Health Service

NI- Northern Ireland

PSS- Personal Social Services

PSHE - Personal, social, health and economic education

STAMPP- Steps Towards Alcohol Misuse Prevention Programme

SHAHRP- Australian School Health and Alcohol Harm Reduction Project

UK - United Kingdom

WTP - Willingness-to-pay

Ethics approval

The research was approved by Liverpool John Moores University Research Ethics Committee (11/HEA/097). Consent was obtained from school head-teachers/principals before randomisation. Consent from participants and their parents/guardians was obtained after randomisation.

Participant consent: obtained

Competing interests: No personal competing interests declared. The sponsor University (LJMU) received and administered a payment from the alcohol industry for printing of student workbooks in the Glasgow trial site only. Percy reported that he has previously received funding from the European Foundation of Alcohol Research (ERAB) in relation to the development of statistical models for longitudinal data (2008-2010). Foxcroft reported that his Department has previously received funding from the alcohol industry for unrelated prevention programme training work. Sumnall reported that his Department has previously received

For peer review only

funding from the alcohol industry (indirectly via the industry funded Drinkaware charity) for unrelated primary research.

Data: The datasets generated during and/or analysed during the current study are not yet publicly available due to the authors undertaking additional analyses and follow-on studies, but are available from the corresponding author on reasonable request.

Patient and Public Involvement

Pupils participating in this trial were not involved in its design. The research questions and outcome measures used in this study were not directly informed by pupils' priorities, experience, and preferences. They were partly aligned to the funding call of the grant awarding body. However, this trial followed an earlier pilot study in Northern Ireland that adapted and evaluated the Australian School Health and Alcohol Harm Reduction Project (SHAHRP) for delivery in the UK, and was informed by pupil and teacher experiences of that research. Education professionals including school head teachers, principals, and subject leads facilitated the participation of pupils in the study, but pupils were not involved in the recruitment and delivery of the study. Study results have been disseminated at a number of special events in the study sites. Experiences of participation in the trial formed part of our process evaluation and this was published as part of our report to funders.[19]

References

1. NHS Digital (2016) Smoking, drinking and drug use among young people. Available from https://files.digital.nhs.uk/07/49FE46/sdd-2016-rep-cor.pdf [Accessed 18/04/19].
2. Scottish Government (2016) Scottish Schools Adolescent Lifestyle and Substance Use Survey (SALSUS): Alcohol Report 2015. Available from https://beta.gov.scot/publications/scottish-schools-adolescent-lifestyle-substance-use-survey-salsus-alcohol-report/pages/3/ [Accessed 18/04/19].
3. Northern Ireland Department of health (2017) Public health NI fact sheet 2017. Available from https://www.health-ni.gov.uk/publications/public-health-ni-fact-sheet-2017 [Accessed 18/04/19]
4. ESPAD Group. ESPAD Report 2015. Results from the European School Survey Project on Alcohol and Other Drugs. Luxembourg, Publications Office of the European Union; 2016
5. World Health Organization. Adolescent alcohol-related behaviours: trends and inequalities in the WHO European Region, 2002–2014. Geneva: World Health Organization; 2018
6. Bonomo Y, Coffey C, Wolfe R, *et al*. Adverse outcomes of alcohol use in adolescents. *Addiction* 2001; 96(10): 1485-1496.

For peer review only

7. Donoghue K, Rose H, Boniface S, *et al*. Alcohol consumption, early-onset drinking, and health-related consequences in adolescents presenting at emergency departments in England. *Journal of Adolescent Health* 2017; 60(4): 438-446.
8. Newbury-Birch D, Gilvarru E, McArdle P, *et al.* Impact of Alcohol Consumption on Young People: A Review of Reviews. London: Department for Children, Schools and Families; 2008.
9. Odgers, CL, Caspi, A, Nagin, DS, *et al*. Is it important to prevent early exposure to drugs and alcohol among adolescents? *Psychological Science* 2008; 19(10): 1037-1044.
10. Degenhardt L, O'Loughlin C, Swift W, *et al*. The persistence of adolescent binge drinking into adulthood: findings from a 15-year prospective cohort study. *BMJ Open* 2013; 3(8): e003015.
11. Percy A, McKay M. The stability of alcohol consumption between age 16 and 26: Evidence from a National Birth Cohort Study. *Journal of Adolescence* 2015; 44: 57-69.
12. James J, Francesconi M. IPR Policy Brief - The cost of binge drinking in the UK. University of Bath; 2015. Available from https://researchportal.bath.ac.uk/en/publications/ipr-policy-brief-the-cost-of-binge-drinking-in-the-uk [Accessed 18/04/19].
13. Levy DT, Miller TR, Cox KC. Costs of Underage Drinking. Washington, DC: US Department of Justice, Office of Justice Programs, Office of Juvenile Justice and Delinquency Prevention; 1999.
14. Institute of Alcohol Studies. The economic impacts of alcohol. June 2016. Available from http://www.ias.org.uk/Alcohol-knowledge-centre/Economic-impacts/Factsheets/Estimates-of-the-cost-of-alcohol.aspx [Accessed 18/04/19)
15. Burton R, Henn C, Lavoie D, *et al*. A rapid evidence review of the effectiveness and cost-effectiveness of alcohol control policies: an English perspective. *Lancet* 2017; 389(10078): 1558-1580.
16. Foxcroft DR, Tsertsvadze A. Universal alcohol misuse prevention programmes for children and adolescents: Cochrane systematic reviews. *Perspect Public Health* 2012 132(3): 128-134.
17. National Institute for Health and Care Excellence (NICE) Alcohol: school-based interventions. London: NICE; 2007.
18. Hill SR, Vale L, Hunter D, *et al*. Economic evaluations of alcohol prevention interventions: Is the evidence sufficient? A review of methodological challenges. *Health policy* 2017; 121(12): 1249-1262.
19. Sumnall H, Agus A, Cole J, *et al*. Steps Towards Alcohol Misuse Prevention Programme (STAMPP): a school- and community-based cluster randomised controlled trial. *Public Health Res* 2017;5(2).
20. McKay M, Agus A, Cole J, *et al*. Steps Towards Alcohol Misuse Prevention Programme (STAMPP): a school-based and community-based cluster randomised controlled trial. *BMJ Open* 2018; Mar 9;8(3):e019722.

For peer review only

21. Harvey SA, McKay M, Sumnall H. Adolescents' reflections on school-based alcohol education in the United Kingdom: education as usual compared with a structured harm reduction intervention. *Journal of Substance Use* 2016; 21(6): 640-645.
22. Hollingworth W, Cohen D, Hawkins J, *et al*. Reducing smoking in adolescents: cost-effectiveness results from the cluster randomized ASSIST (A Stop Smoking In Schools Trial). *Nicotine Tob Res* 2012;14(2):161-8.
23. Mytton J, Ingram J, Manns S, *et al*. The feasibility of using a parenting programme for the prevention of unintentional home injuries in the under-fives: a cluster randomised controlled trial. *Health Technol Assess* 2014; 18(3):1-184.
24. Lohan M, Aventin Á, Maguire L, *et al*. Increasing boys' and girls' intentions to avoid teenage pregnancy: a cluster randomised controlled feasibility trial of an interactive video drama-based intervention in post-primary schools in Northern Ireland. Public *Health Res* 2017; 5(1).
25. Beecham J, Knapp M. Costing psychiatric interventions. In Measuring Mental Health Needs (eds G. Thornicroft, C.R.Brewin & J.Wing), pp.163-183. London:Gaskell; 1992.
26. Knapp M, Scott S, Davies J. The cost of antisocial behaviour in younger children. *Clinical Child Psychology and Psychiatry* 1999; 4: 457-473.
27. Department of Health. NHS Reference Costs 2013-14, Department of Health, London. 2014.
28. Curtis L, ed. Unit Costs of Health and Social Care 2014. Canterbury: University of Kent: Personal Social Services Research Unit. 2014.
29. National Society for Prevention of Cruelty to Children. Piece by Piece. Annual report and account 2014/15. 2015. Available from https://www.nspcc.org.uk/globalassets/documents/annual-reports/nspcc-annual-report-2014-15.pdf [Accessed 18/04/19].
30. Department of Education Northern Ireland. Circular 2014/13 - Teachers' pay and allowances from 1 September 2013. 2014. Available from https://www.deni.gov.uk/publications/circular-201413-teachers-pay-and-allowances-1-september-2013 [Accessed 18/04/19].
31. Johannesson M, Weinstein S. On the decision rules of cost- effectiveness analysis. J Health Econ 1993;12:459–67
32. National Institute for Health and Care Excellence. Guide to the methods of technology appraisal 2013. London: NICE; 2013. Available from https://www.nice.org.uk/process/pmg9/resources/guide-to-the-methods-of-technology-appraisal-2013-pdf-2007975843781 [Access 18/04/19].
33. National Institute for Health and Care Excellence. Methods for the Development of NICE Public Health Guidance. London: NICE; 2012. Available from https://www.nice.org.uk/process/pmg4/resources/methods-for-the-development-of-nice-public-health-guidance-third-edition-pdf-2007967445701
34. Glick HA, Doshi JA, Sonnad SS, *et al*. Economic evaluation in clinical trials. Oxford: Oxford University Press; 2007.
35. Drummond M, Sculpher M, Torrance G, *et al*. Methods for the Economic Evaluation of Health Care Programmes. Third Edition. OUP. Oxford; 2005.

For peer review only

36. McBride N, Farringdon F, Midford R. *et al*. Harm minimization in school drug education: final results of the School Health and Alcohol Harm Reduction Project (SHAHRP). A*ddiction* 2004; 99 (3): 278-291.
37. Eisen M, Zellman GL, Murray DM. Evaluating the Lion's-Quest "Skills for Adolescence" drug education program. Second-year behavior outcomes. *Addictive Behaviors* 2003; 28 (5): 883-897.
38. Werch CE., Pappas DM., Carlson JM, *et al*. Evaluation of a brief alcohol prevention program for urban school youth. *American Journal of Health Behavior* 2000; 24 (2): 120-131.
39. Miller, TR, Hendrie, D. Substance Abuse Prevention Dollars and Cents: A Cost-Benefit Analysis. Center for Substance Abuse Prevention, Substance Abuse and Mental Health Services Administration; 2008.
40. Leigh BC, Gillmore MR, Morrison DM. Comparison of diary and retrospective measures for recording alcohol consumption and sexual activity. *J Clin Epidemiol* 1998; 51:119–27.
41. Lintonen T, Ahlström S, Metso L. The reliability of self-reported drinking in adolescence. *Alcohol Alcohol* 2004; 39:362–8.
42. Percy A, McAlister S, Higgins K, *et al.* Response consistency in young adolescents' drug use self-reports: a recanting rate analysis. *Addiction* 2005, 100(2), 189-196.
43. Percy A, Agus A, Cole J, *et al*. Recanting of Previous Reports of Alcohol Consumption within a Large-Scale Clustered Randomised Control Trial. *Prevention Science* 2019 Jan 14
44. Claxton K. The irrelevance of inference: a decision-making approach to the stochastic evaluation of health care technologies. *J Health Econ* 1999; 18(3):341-64.
45. Rehm J., Baliunas D, Borges GL, et al. The relation between different dimensions of alcohol consumption and burden of disease: an overview. *Addiction* 2010; 105(5), 817-843.
46. Cookson R, Mirelman, AJ, Griffin S, *et al*. Using Cost-Effectiveness Analysis to Address Health Equity Concerns. *Value in Health* 2017; 20(2), 206-212.

For peer review only

616 Table 1 Unit costs (UK £) of public sector services

Service	Unit Cost	Source
**Education**		
School nurse	50.00	Unit Costs of Health and Social Care 2014[29] p.85
School counsellor/guidance teacher	35.02	Department of Education Northern Ireland[25] (per 1 hour of teacher time, point 2 of upper pay scale)
Intervention teacher	25.89	Department of Education Northern Ireland[25] (per 1 hour of teacher time, point 3 of main pay scale)
Educational psychologist	41.00	Unit Costs of Health and Social Care 2014[29] p.156
Education welfare officer/ home-school liaison officer	27.00	Unit Costs of Health and Social Care 2014[29] p.155
**Health**		
GP surgery visit	46.00	Unit Costs of Health and Social Care 2014[29] p.195
GP out of hours	115.00	Unit Costs of Health and Social Care 2014[29] p.191 (home visit unit cost assumed as above)
Nurse (other than school nurse)	13.70	Unit Costs of Health and Social Care 2014[29] p.192 (per 15.5 minute surgery consultation)
Hospital appointment	109.00	Unit Costs of Health and Social Care 2014[29]p.111
Accident & Emergency	233.00	Unit Costs of Health and Social Care 2014[29] p.111 (see and treat and convey)
Overnight hospital stay	658.33	NHS Reference Costs 2013-14[29] (weighted average length of stay and cost of paediatric non-elective long stays)
Psychologist	50.00	Unit Costs of Health and Social Care 2014[29] p.51
Counsellor (other than at school)	50.00	Unit Costs of Health and Social Care 2014[29] p.51
Social worker	79.00	Unit Costs of Health and Social Care 2014[29] p.206 (per 1 hour including travel)
Telephone help-line	3.99	National Society for the Prevention of Cruelty to Children Annual Report and Account 2014/2015[30] cost per call to Childline deflated to 2013–14
**Criminal Justice**		
Youth justice service	84.00	Unit Costs of Health and Social Care 2014[29] p.224 (face-to-face contact)
Police service	325.00	Unit Costs of Health and Social Care 2014[29] p.149

617

618

For peer review only

Table 2 Total cost to deliver STAMPP

**Stage 1 Planning and preparation for delivery**	Total Cost (£)
Materials	£6,694
Training	£38,079
**Stage 1 subtotal**	**£44,773**
**Stage 2 Delivery**	
Teaching	£27,877
Facilitator (for parental component)	£13,250
**Stage 2 subtotal**	**£41,127**
**STAMPP Total Cost**	**£85,900**
Mean cost/school[1]	**£818**
Mean cost/pupil[2]	**£15**

[1]based on 5749 pupils at baseline and 192 classes
[2]based on 105 schools

For peer review only

626 Table 3 Costs (£) at 33 months, by group.

	**Intervention** n=4189	**Education as normal** n=4037	**Difference (95% CI)**
Costs (£; mean; 95% CI)			
Education	287.46 (247.33, 327.58)	284.81 (244.20, 325.42)	2.65 (-54.29, 59.60)
Health	1839.07 (1564.87, 2113.26)	1906.55 (1627.59, 51)	-67.48 (-460.16, 325.19)
Criminal	128.16 (76.67, 179.66)	101.89 (49.43, 154.35)	26.27 (-47.55, 100.10)
Total Public Service Costs	2260.47 (1950.23, 2570.72)	2292.66 (1977.10, 2608.22)	-32.19 (-476.38, 412.00)

627 Costs discounted at 3.5%. Values are mean (95% confidence interval) adjusted for baseline covariates and
628 clustering. n, number analysed.
629

For peer review only

630 Table 4 Proportion of pupils reporting any heavy drinking episode in the previous 30 days at
631 33 months by group.

**Intervention** n=4189	**Education as normal** n=4037	Difference in effect (proportion of pupils reporting a heavy drinking episode in previous 30 days *avoided*) **(95% CI)***
0.15 (0.14, 0.17)	0.23 (0.21, 0.25)	0.08 (0.06, 0.09)

632 Values are proportion (95% confidence interval) adjusted for baseline covariates and clustering. n= number
633 analysed
634 * Confidence interval based on 1000 bootstrap resamples
635
636

For peer review only

Table 5 Results of the primary cost-effectiveness analysis and sensitivity analyses.

Analysis	Intervention, number analysed (% total)	Education as normal, number analysed (% total)	Difference in total costs (£) (intervention + total public service costs)	Difference in effect (proportion of pupils reporting a heavy drinking episode in previous 30 days *avoided*)
Primary analysis at 33 months	4189 (65.7)	4037 (63.5)	-17.19 (-402.84, 368.46)	0.08 (0.06, 0.09)
Sensitivity analysis for primary cost-effectiveness analysis at 33 months				
Multiple imputation for missing cost and outcome data	6379 (100)	6359 (100)	34.10 (-299.44, 367.44)	0.08 (0.07, 0.09)
Discounting costs and outcome at 1.5%	4189 (65.7)	4037 (63.5)	-17.63 (-410.62, 375.36)	0.07 (0.06, 0.09)
Adjustment of costs and outcome data for cluster only	4189 (65.7)	4037 (63.5)	-49.23 (-419.52, 321.07)	0.08 (0.06, 0.10)
5% increase in costs	4189 (65.7)	4037 (63.5)	-18.05 (-422.98, 386.88)	0.07 (0.06, 0.09)
5% decrease in costs	4189 (65.7)	4037 (63.5)	-16.33 (-382.70, 350.04)	0.07 (0.06, 0.09)

Total participants in the study= 12738 (Intervention=6379, Education as Normal=6359).

Figure 1 Cost-effectiveness plane for the primary cost-effectiveness analysis showing bootstrapped replications of mean incremental costs and pupils experiencing heavy drinking-avoided

Figure 2 Cost effectiveness acceptability curve showing the probability of STAMPP being cost-effective compared to education as normal.

For peer review only

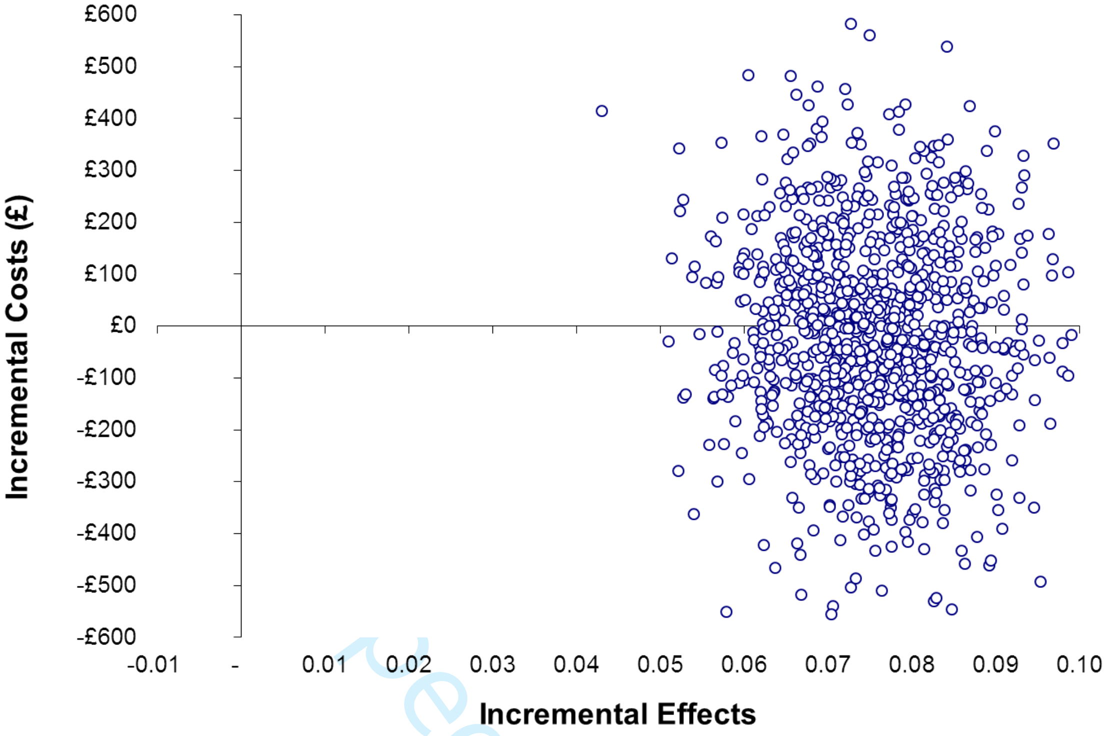

Figure 1 Cost-effectiveness plane for the primary cost-effectiveness analysis showing bootstrapped replications of mean incremental costs and pupils experiencing heavy drinking-avoided.

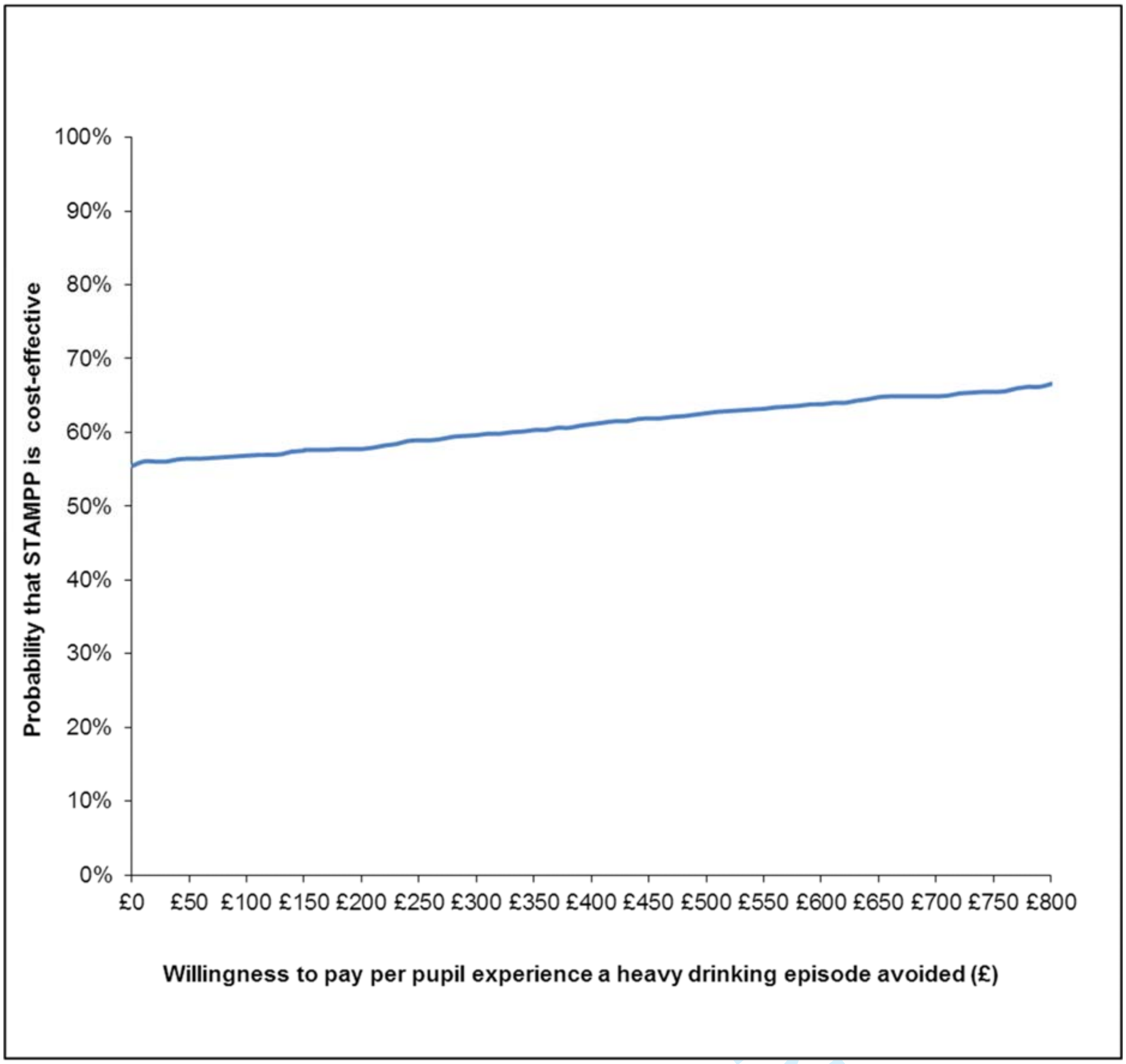

Figure 2 Cost effectiveness acceptability curve showing the probability of STAMPP being cost-effective compared to education as normal.

Online Supplementary File Cost-effectiveness of a combined classroom curriculum and parental intervention: economic evaluation of data from the Steps Towards Alcohol Misuse Prevention Programme (STAMPP) cluster randomised controlled trial (Agus et al).

Costing the intervention

Stage 1 resources in STAMPP covered; recurring costs associated with printing/copying the intervention materials (teacher manual, pupil work book, parent/guardian information sheets, CD-ROMs) and the delivery of teacher training (trainer fees, venue, catering and teacher cover). No costs associated with the purchasing and licensing of the STAMPP intervention are foreseen and so are not included here. Stage 2 resources covered recurring costs associated with teacher time input for delivery of Phase 1 and Phase 2 of the programme. The cost of teaching time was included, even though the intervention was delivered within normal working hours and so would not have been an extra cost to the public sector. In doing so we captured the opportunity cost of the benefits to pupils which were foregone by STAMPP being delivered instead of other lessons.

Resources used during stages 1 and 2 were recorded retrospectively by the project team and costs were obtained from invoices when available. The costs associated with printing the intervention materials were not available as these resources were present at the start of the trial. We therefore obtained printing quotations and deflated it to the appropriate price year using the Consumer Price Index.[1] The Local Authority in Glasgow/Inverclyde covered all the costs associated with the training of the teachers including teacher cover, venue and catering and so these were also not available. We therefore used the costs that were available for NI and applied it to Scotland. The majority of teachers delivering the intervention were at a junior level, thus we used point 3 on the Main Pay Scale[2] including 24% for employers' costs.

Stage 1 costs were added together to get a total cost. Stage 2 costs relating to the delivery of the intervention at a class level were multiplied by the total number of classes that received intervention to get a total cost. This was estimated by dividing the number of pupils receiving the intervention at baseline (n=5749) by 30, which was used as an estimate of the maximum number of pupils per class. Stage 2 costs relating to the delivery of the parental component were calculated by multiplying the number of schools by the costs to deliver one session. Stage

For peer review only

1 and Stage 2 costs were totalled and divided by the number of pupils receiving the intervention at baseline to obtain the mean cost per pupil.

1. Office for National Statistics. (2015). CPI annual percentage change: 1989 to 2015. Retrieved from http://www.ons.gov.uk/ons/datasets-and-tables/data-selector.html?cdid=D7G7&dataset=mm23&table-id=1.2 (accessed Dec 9 2015)
2. Department of Education Northern Ireland. Circular 2014/13 - Teachers' pay and allowances from 1 September 2013. 2014. Available from https://www.deni.gov.uk/publications/circular-201413-teachers-pay-and-allowances-1-september-2013 (accessed Dec 9 2015)

Table S1 Breakdown of missing cost and/outcome data by group

Missing data	Intervention n=2190 [%]	Education as normal n=2322 [%]	Total n=4512 [%]
Outcome	1219 [57]	1286 [55]	2505 [56]
Cost	2123 [97]	2256 [97]	4379 [97]
Both	1152 [53]	1220 [53]	2372 [53]

Table S2 Intervention resource use and costs

**Resource use**	**Unit cost (£)**	**Number of units**	**Total cost (£)**	**Cost details**
**Stage 1 Planning and preparation for delivery**				
**Intervention materials**				
Phase 1 Teacher Manual	n/a	145	240	16 pages
Phase 2 Teacher Manual	n/a	145	293	22 pages
Phase 1 Pupil Workbook	n/a	5749	2623	20 pages
Phase 2 Pupil Workbook	n/a	5749	2623	20 pages
Parent & Guardian Information Sheet	n/a	5749	255	single-sided page
Parent & Guardian Information Sheet (follow up)	n/a	5749	255	single-sided page
Phase 1 CD-ROM	1.40	145	203	cost of duplication
Phase 2 CD-ROM	1.40	145	203	cost of duplication
**Training of the teachers (Phase 1)**				

For peer review only - http://bmjopen.bmj.com/site/about/guidelines.xhtml

Northern Ireland				
Training	500.00	3	1500	Training delivered by a non-government organisation facilitator (£500 per day)
Teachers	161.79	107	17312	Based on 1 day (6.5 hours) teaching cover for a junior teacher at £24.89/hour
Location & associated cost (e.g. room hire)	25.00	107	2675	Based on local hotel costs including food & refreshments per head
Scotland				
Training	500.00	2	1000	Training delivered by a non-government organisation facilitator (£500 per day)
Teachers	161.79	38	6148	Based on 1 day (6.5 hours) teaching cover for a junior teacher at £24.89/hour
Location & associated cost (e.g. room hire)	25.00	38	950	Local hotel costs including food & refreshments (£25/head)
**Training of the teachers (Phase 2)**				
Northern Ireland				
Training sessions	500.00	3	1500	Training delivered by a non-government organisation facilitator (£500 per day)
Teachers	24.89	83	2066	based on 1 day (6.5 hours) teaching cover for a junior teacher
Location & associated cost (e.g. room hire)	28.78	83	2389	Local hotel costs including food & refreshments (£28.78/head)
Scotland				
Trainer	500.00	1	500	Training delivered by a non-government organisation facilitator (£500 per day)
Teachers	24.89	38	946	based on 1 day (6.5 hours) teaching cover for a junior teacher
Location & associated cost (e.g. room hire)	28.78	38	1094	Local hotel costs including food & refreshments (£28.78/head)
**Stage 2 Delivery**				
Phase 1 Teacher time input	14.52	6	87	Based on 35 minute lessons
Phase 2 Teacher time input	14.52	4	58	Based on 35 minute lessons

For peer review only - http://bmjopen.bmj.com/site/about/guidelines.xhtml

Parental component delivery	250.00	53	13250	Delivered by a non-government organisation facilitator (£500 per day)

For peer review only

Use of public sector services by all pupils with available data at each of the time points.

Table S3 Public sector service by treatment arm from 6 to 12 months. Values are number (percentages) of pupils using the service and mean (SD) use.

Service	Intervention (n=4189)		Education as normal (n=4037)	
	**Number using the service (%)**	**Mean (SD)**	**Number using the service (%)**	**Mean (SD)**
**Education**				
School nurse	1792 (42.8)	0.9 (3.0)	1518 (37.6)	0.9 (3.4)
School counsellor/guidance teacher	642 (15.3)	0.5 (3.5)	646 (16.0)	0.4 (1.8)
Educational psychologist	71 (1.7)	0.0 (0.8)	84 (2.1)	0.0 (0.4)
Education welfare officer/ home-school liaison officer	59 (1.4)	0.0 (0.4)	89 (2.2)	0.0 (0.9)
**Health**				
GP surgery visit	2033 (48.5)	1.1 (5.0)	1791 (44.4)	0.9 (1.8)
GP out of hours	521 (12.4)	0.2 (0.9)	423 (10.5)	0.2 (0.8)
Nurse (other than school nurse)	839 (20.0)	0.3 (0.9)	705 (17.5)	0.3 (0.8)
Hospital appointment	1338 (31.9)	0.6 (1.5)	1200 (29.7)	0.6 (2.1)
Accident & Emergency	827 (19.7)	0.3 (0.7)	737 (18.3)	0.3 (1.1)
Overnight hospital stay	270 (6.5)	0.2 (2.0)	261 (6.5)	0.3 (3.7)
Psychologist	72 (1.7)	0.0 (0.8)	81 (2.0)	0.0 (0.5)
Counsellor (other than at school)	76 (1.8)	0.1 (0.9)	65 (1.6)	0.1 (1.4)
Social worker	142 (3.4)	0.1 (1.7)	112 (2.8)	0.1 (1.6)
Telephone help-line	30 (0.7)	0.0 (0.4)	46 (1.1)	0.0 (0.4)
**Criminal Justice**				
Youth justice service	32 (0.8)	0.0 (0.6)	33 (0.8)	0.0 (0.3)
Police service	108 (2.6)	0.0 (0.4)	120 (3.0)	0.0 (0.4)

For peer review only

Table S4 Public sector service by treatment arm from 18 to 24 months. Values are number (percentages) of pupils using the service and mean (SD) use.

Service	Intervention (n=4189)		Education as normal (n=4037)	
	Number using the service (%)	Mean (SD)	Number using the service (%)	Mean (SD)
**Education**				
School nurse	1403 (33.5)	0.7 (2.2)	1325 (32.8)	0.7 (3.4)
School counsellor/guidance teacher	567 (13.5)	0.4 (3.0)	608 (15.1)	0.5 (3.3)
Educational psychologist	62 (1.5)	0.1 (1.9)	42 (1.0)	0.0 (0.5)
Education welfare officer/ home-school liaison officer	84 (2.0)	0.1 (1.3)	55 (1.4)	0.0 (0.5)
**Health**				
GP surgery visit	2013 (48.1)	0.9 (2.2)	1978 (49.0)	0.9 (1.4)
GP out of hours	498 (11.9)	0.2 (1.8)	462 (11.4)	0.2 (0.6)
Nurse (other than school nurse)	768 (18.3)	0.3 (2.2)	752 (18.6)	0.3 (1.0)
Hospital appointment	1269 (30.3)	0.6 (2.7)	1269 (31.4)	0.6 (0.8)
Accident & Emergency	764 (18.2)	0.3 (2.3)	810 (20.1)	0.3 (0.8)
Overnight hospital stay	245 (5.9)	0.3 (3.3)	266 (6.6)	0.3 (7.3)
Psychologist	86 (2.1)	0.1 (2.6)	91 (2.3)	0.1 (1.0)
Counsellor (other than at school)	111 (2.7)	0.1 (2.8)	104 (2.6)	0.1 (1.1)
Social worker	137 (3.3)	0.6 (3.3)	132 (3.3)	0.1 (1.2)
Telephone help-line	42 (1.0)	0.1 (3.0)	48 (1.2)	0.1 (0.9)
**Criminal Justice**				
Youth justice service	31 (0.7)	0.1 (3.2)	30 (0.7)	0.0 (1.0)
Police service	144 (3.4)	0.1 (3.3)	129 (3.2)	0.1 (1.6)

For peer review only

Table S5 Public sector service by treatment arm from 27 to 33 months. Values are number (percentages) of pupils using the service and mean (SD) use.

Service	Intervention (n=4189)		Education as normal (n=4037)	
	Number using the service (%)	Mean (SD)	Number using the service (%)	Mean (SD)
**Education**				
School nurse	1195 (28.5)	0.6 (2.2)	1094 (27.1)	0.5 (1.4)
School counsellor/guidance teacher	558 (13.3)	0.6 (3.7)	560 (13.9)	0.6 (3.8)
Educational psychologist	41 (0.98)	0.0 (0.4)	39 (1.0)	0.0 (0.4)
Education welfare officer/ home-school liaison officer	64 (1.5)	0.1 (4.5)	56 (1.4)	0.0 (0.5)
**Health Sector**				
GP surgery visit	1943 (46.4)	1.0 (1.8)	1790 (44.3)	1.0 (2.2)
GP out of hours	399 (9.5)	0.1 (0.6)	350 (8.7)	0.1 (0.7)
Nurse (other than school nurse)	622 (14.9)	0.3 (0.9)	593 (14.7)	0.3 (1.0)
Hospital appointment	1073 (25.6)	0.6 (1.7)	975 (24.2)	0.5 (2.0)
Accident & Emergency	635 (15.2)	0.2 (0.6)	626 (15.5)	0.2 (1.0)
Overnight hospital stay	169 (4.0)	0.1 (1.0)	161 (4.0)	0.1 (1.0)
Psychologist	85 (2.0)	0.1 (1.5)	73 (1.8)	0.1 (0.8)
Counsellor (other than at school)	96 (2.29)	0.2 (1.6)	98 (2.4)	0.1 (1.6)
Social worker	85 (2.03)	0.1 (1.3)	108 (2.7)	0.1 (1.1)
Telephone help-line	43 (1.0)	0.0 (0.6)	39 (1.0)	0.0 (0.4)
**Criminal Justice Service**				
Youth justice service	10 (0.2)	0.0 (0.3)	18 (0.5)	0.0 (0.3)
Police service	97 (2.3)	0.1 (0.5)	111 (2.8)	0.0 (0.6)

For peer review only

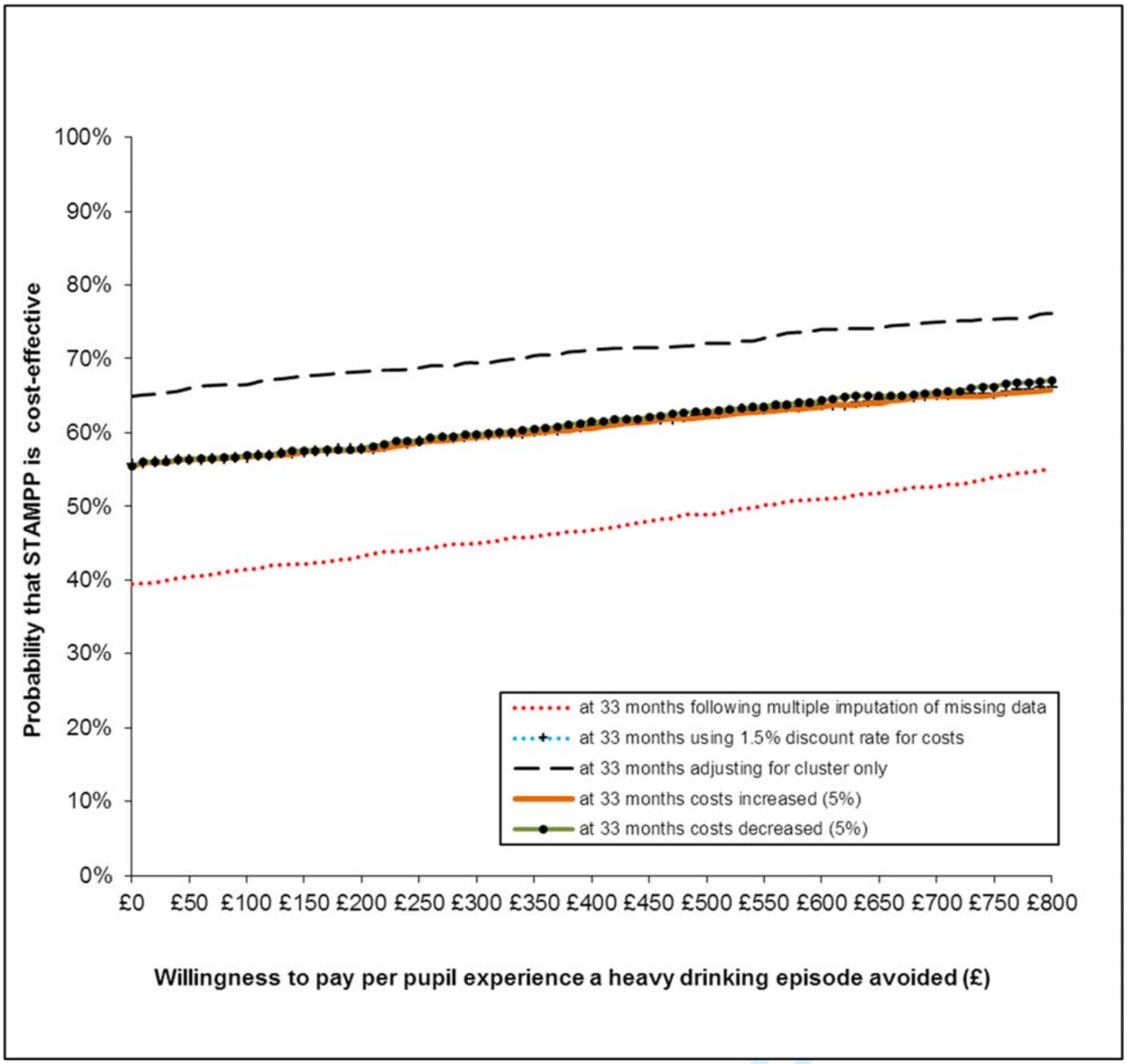

Figure S1 Cost effectiveness acceptability curves showing the probability of STAMPP being cost-effective compared to usual education for the sensitivity analyses.

Table S6 Baseline characteristics of pupils' with complete and non-complete health economic data by intervention arm. Values are counts [percentage] or mean [95% Confidence interval]

Baseline characteristics	Complete data (included in cost-effectiveness analysis)			Incomplete data (excluded from cost-effectiveness analysis)		
	Intervention n=4189 [%]	Education as normal n=4037[%]	Total n=8226 [%]	Intervention n=2190 [%]	Education as normal n=2322[%]	Total n=4512 [%]
School location						
NI	2764 [66]	2637 [65]	5401 [66]	1085 [50]	1256 [54]	2341 [52]
Scotland	1425 [34]	1400 [35]	2825 [34]	1105 [50]	1066 [46]	2171 [48]
*School free school meal category**						
Low	1720 [41]	1442 [36]	3162 [38]	617 [28]	509 [22]	1126 [25]
Moderate	1672 [40]	1469 [36]	3141 [38]	880 [40]	991 [43]	1871 [41]
High	797 [19]	1126 [28]	1923 [23]	693 [32]	822 [35]	1515 [34]
School type						
Co-education	3353 [80]	3229 [80]	6582 [80]	1910 [87]	1989 [86]	3899 [86]
Girls	541 [13]	394 [10]	935 [11]	175 [8]	114 [5]	289 [6]
Boys	295 [7]	414 [10]	709 [9]	105 [5]	219 [9]	324 [7]
Pupil reported heavy episodic drinking						
Yes	198 [5]	213 [5]	411 [5]	233 [11]	219 [9]	452 [10]
No	3588 [86]	3319 [82]	6907 [84]	1673 [76]	1763 [76]	3436 [76]
Missing	403 [10]	505 [13]	908 [11]	284 [13]	340 [15]	624 [14]

* Percentage of n (n=Free school meal provision- Low; 0-15.4%, Moderate; 15.5-30.4%, High; 30.5% and above

Table S7 Public service costs (£UK) in the 6 months pre-baseline (reported at baseline).

Baseline characteristic	Complete data (included in cost-effectiveness analysis)			Incomplete data (excluded from cost-effectiveness analysis)		
	Intervention n=4189	Education as normal n=4037	Total n=8226	Intervention n=2190	Education as normal n=2322	Total n=4512
Mean [95% CI]	620 [481-759]	631 [487-774]	625 [525-725]	667 [572-761]	698 [612-783]	682 [619, 746]

For peer review only

Missing (n; %)	371 [9]	484 [12]	855 [10]	259 [12]	308 13]	567 [13]

For peer review only

Alternative intervention costs

We explored two alternative scenarios for delivering STAMPP- 1) the removal of the parental component making the intervention classroom based only, 2) the removal of the parental; component and the the costs associated with teaching time (to reflect STAMPP being incorporated in to the curriculum).

Table S8 Total cost to deliver STAMPP

**Stage 1 Planning and preparation for delivery**	Total Cost (£)
Materials	£6,694
Training	£38,079
**Stage 1 subtotal**	**£44,773**
**Stage 2 Delivery**	
Teaching	£27,877
Facilitator (for parental component)	£13,250
**Stage 2 subtotal**	**£41,127**
**STAMPP Total Cost**	**£85,900**
Mean cost/school[1]	**£818**
Mean cost/pupil[2]	**£15**
**Classroom component only- Total Cost[3]**	**£72,650**
Mean cost/school[1]	**£692**
Mean cost/pupil[2]	**£13**
**Materials and teacher training only- Total Cost[4]**	**£44,773**
Mean cost/school[1]	**£426**
Mean cost/pupil[2]	**£8**

[1]based on 5749 pupils at baseline and 192 classes

[2]based on 105 schools

[3]excluding parental component cost of £13,250 for facilitator

[4] based on Stage 1 costs only

For peer review only - http://bmjopen.bmj.com/site/about/guidelines.xhtml

Consolidated Health Economic Evaluation Reporting Standards (CHEERS) Checklist

Items to include when reporting economic evaluations of health interventions

Section/Topic	Item No	Recommendation	Reported on page No / Line No
**Title and abstract**			
Title	1	Identify the study as an economic evaluation or use more specific terms such as "cost-effectiveness analysis", and describe the interventions compared.	**Line 1**
Abstract	2	Provide a structured summary of objectives, perspective, setting, methods (including study design and inputs), results (including base case and uncertainty analyses), and conclusions.	**Lines 6-36**
**Introduction**			
Background and objectives	3	Provide an explicit statement of the broader context for the study.	**Lines 63-66**
		Present the study question and its relevance for health policy or practice decisions.	**Lines 90-91**
**Methods**			
Target population and subgroups	4	Describe characteristics of the base case population and subgroups analysed, including why they were chosen.	**Lines 94-97**
Setting and location	5	State relevant aspects of the system(s) in which the decision(s) need(s) to be made.	**Lines 95 -96**
Study perspective	6	Describe the perspective of the study and relate this to the costs being evaluated.	**Lines 99-101**
Comparators	7	Describe the interventions or strategies being compared and state why they were chosen.	**Lines 94- details of intervention in main trial paper**
Time horizon	8	State the time horizon(s) over which costs and consequences are being evaluated and say why appropriate.	**Line 99**
Discount rate	9	Report the choice of discount rate(s) used for costs and outcomes and say why appropriate.	**Lines 218-219, lines 233-234**
Choice of health outcomes	10	Describe what outcomes were used as the measure(s) of benefit in the evaluation and their relevance for the type of analysis performed.	**Lines 104-108**
Measurement of effectiveness	11a	*Single study-based estimates:* Describe fully the design features of the single effectiveness study and why the single study was a sufficient source of clinical effectiveness data.	**Full details of trial in main paper**
	11b	*Synthesis-based estimates:* Describe fully the methods used for identification of included studies and synthesis of clinical effectiveness data.	**n/a**
Measurement and valuation of preference-	12	If applicable, describe the population and methods used to elicit preferences for outcomes.	**n/a**

For peer review only

based outcomes			
Estimating resources and costs	13a	*Single study-based economic evaluation:* Describe approaches used to estimate resource use associated with the alternative interventions. Describe primary or secondary research methods for valuing each resource item in terms of its unit cost. Describe any adjustments made to approximate to opportunity costs.	**Line 112-188**
	13b	*Model-based economic evaluation:* Describe approaches and data sources used to estimate resource use associated with model health states. Describe primary or secondary research methods for valuing each resource item in terms of its unit cost. Describe any adjustments made to approximate to opportunity costs.	**n/a**
Currency, price data, and conversion	14	Report the dates of the estimated resource quantities and unit costs. Describe methods for adjusting estimated unit costs to the year of reported costs if necessary. Describe methods for converting costs into a common currency base and the exchange rate.	**Lines 177-178, Table 1**
Choice of model	15	Describe and give reasons for the specific type of decision-analytical model used. Providing a figure to show model structure is strongly recommended.	**n/a**
Assumptions	16	Describe all structural or other assumptions underpinning the decision-analytical model.	**n/a**
Analytic methods	17	Describe all analytical methods supporting the evaluation. This could include methods for dealing with skewed, missing, or censored data; extrapolation methods; methods for pooling data; approaches to validate or make adjustments (such as half cycle corrections) to a model; and methods for handling population heterogeneity and uncertainty.	**Lines 191-240**
**Results**			
Study parameters	18	Report the values, ranges, references, and, if used, probability distributions for all parameters. Report reasons or sources for distributions used to represent uncertainty where appropriate. Providing a table to show the input values is strongly recommended.	**n/a**
Incremental costs and outcomes	19	For each intervention, report mean values for the main categories of estimated costs and outcomes of interest, as well as mean differences between the comparator groups. If applicable, report incremental cost-effectiveness ratios.	**Table 2, Table 3, Table 4, Table 5**
Characterizing uncertainty	20a	*Single study-based economic evaluation:* Describe the effects of sampling uncertainty for the estimated incremental cost and incremental effectiveness parameters, together with the impact of methodological assumptions (such as discount rate, study perspective).	**Table 5 line 289-307**
	20b	*Model-based economic evaluation:* Describe the effects on the results of uncertainty for all input parameters, and uncertainty related to the structure of the model and assumptions.	**n/a**
Characterizing heterogeneity	21	If applicable, report differences in costs, outcomes, or cost-effectiveness that can be explained by variations between subgroups of patients with different baseline characteristics or other observed variability in effects that are not reducible by more information.	**n/a**
**Discussion**			
Study findings, limitations, generalizability, and current knowledge	22	Summarise key study findings and describe how they support the conclusions reached. Discuss limitations and the generalisability of the findings and how the findings fit with current knowledge.	**Lines 311-402**
**Other**			
Source of funding	23	Describe how the study was funded and the role of the funder in the identification, design, conduct, and reporting of the analysis. Describe other non-monetary sources of support.	**Lines 421-426**
Conflicts of interest	24	Describe any potential for conflict of interest of study contributors in accordance with journal policy. In the absence of a journal policy, we recommend authors comply with International Committee of Medical Journal Editors recommendations.	**Lines 458-465**

For peer review only

For peer review only
